# Ambient and household PM_2.5_ pollution and adverse perinatal outcomes: A meta-regression and analysis of attributable global burden for 204 countries and territories

**DOI:** 10.1371/journal.pmed.1003718

**Published:** 2021-09-28

**Authors:** Rakesh Ghosh, Kate Causey, Katrin Burkart, Sara Wozniak, Aaron Cohen, Michael Brauer

**Affiliations:** 1 Institute for Global Health Sciences, University of California, San Francisco, San Francisco, California, United States of America; 2 Institute for Health Metrics and Evaluation, University of Washington, Seattle, Washington, United States of America; 3 Boston University School of Public Health, Boston, Massachusetts, United States of America; 4 Health Effects Institute, Boston, Massachusetts, United States of America; 5 School of Population and Public Health, The University of British Columbia, Vancouver, British Columbia, Canada; The Hospital for Sick Children, CANADA

## Abstract

**Background:**

Particulate matter <2.5 micrometer (PM_2.5_) is associated with adverse perinatal outcomes, but the impact on disease burden mediated by this pathway has not previously been included in the Global Burden of Disease (GBD), Mortality, Injuries, and Risk Factors studies. We estimated the global burden of low birth weight (LBW) and preterm birth (PTB) and impacts on reduced birth weight and gestational age (GA), attributable to ambient and household PM_2.5_ pollution in 2019.

**Methods and findings:**

We searched PubMed, Embase, and Web of Science for peer-reviewed articles in English. Study quality was assessed using 2 tools: (1) Agency for Healthcare Research and Quality checklist; and (2) National Institute of Environmental Health Sciences (NIEHS) risk of bias questions. We conducted a meta-regression (MR) to quantify the risk of PM_2.5_ on birth weight and GA. The MR, based on a systematic review (SR) of articles published through April 4, 2021, and resulting uncertainty intervals (UIs) accounted for unexplained between-study heterogeneity. Separate nonlinear relationships relating exposure to risk were generated for each outcome and applied in the burden estimation.

The MR included 44, 40, and 40 birth weight, LBW, and PTB studies, respectively. Majority of the studies were of retrospective cohort design and primarily from North America, Europe, and Australia. A few recent studies were from China, India, sub-Saharan Africa, and South America. Pooled estimates indicated 22 grams (95% UI: 12, 32) lower birth weight, 11% greater risk of LBW (1.11, 95% UI: 1.07, 1.16), and 12% greater risk of PTB (1.12, 95% UI: 1.06, 1.19), per 10 μg/m^3^ increment in ambient PM_2.5_. We estimated a global population–weighted mean lowering of 89 grams (95% UI: 88, 89) of birth weight and 3.4 weeks (95% UI: 3.4, 3.4) of GA in 2019, attributable to total PM_2.5_. Globally, an estimated 15.6% (95% UI: 15.6, 15.7) of all LBW and 35.7% (95% UI: 35.6, 35.9) of all PTB infants were attributable to total PM_2.5_, equivalent to 2,761,720 (95% UI: 2,746,713 to 2,776,722) and 5,870,103 (95% UI: 5,848,046 to 5,892,166) infants in 2019, respectively. About one-third of the total PM_2.5_ burden for LBW and PTB could be attributable to ambient exposure, with household air pollution (HAP) dominating in low-income countries. The findings should be viewed in light of some limitations such as heterogeneity between studies including size, exposure levels, exposure assessment method, and adjustment for confounding. Furthermore, studies did not separate the direct effect of PM_2.5_ on birth weight from that mediated through GA. As a consequence, the pooled risk estimates in the MR and likewise the global burden may have been underestimated.

**Conclusions:**

Ambient and household PM_2.5_ were associated with reduced birth weight and GA, which are, in turn, associated with neonatal and infant mortality, particularly in low- and middle-income countries.

## Abstract 2

Rakesh Ghosh and co-workers report on associations between particulate matter air pollution and adverse perinatal health outcomes.

## Author summary

**Why was this study done?:**

- Air pollution is a leading risk factor for global disease burden, yet its impacts on perinatal outcomes have not previously been included, despite evidence from more than 150 original research articles showing evidence on the adverse effects of exposure to particulate matter <2.5 micrometer (PM_2.5_) during pregnancy.
- Burden assessments conducted to date have used pooled estimates from meta-analyses restricted to ambient PM_2.5_ studies from countries with relatively low levels, with an underlying assumption of a linear concentration–response relationship. However, sufficient evidence from high-pollution settings was not available to support linear extrapolation of risk, likely overestimating the attributable global burden.
- Further, excluding household air pollution (HAP), a major source of PM_2.5_ pollution in many Asian and African countries, does not allow true estimation of the total global burden attributable to PM_2.5_.

**What did the researchers do and find?:**

- To our knowledge, this is the first assessment of global burden that includes 4 perinatal health indicators (birth weight, gestational age [GA], low birth weight [LBW], and preterm birth [PTB]) and includes both ambient and household PM_2.5_ to estimate the complete burden from total PM_2.5_ exposure.
- We used a conservative nonlinear risk relationship generated using both ambient and HAP studies and with uncertainty intervals (UIs) accounting for between-study heterogeneity in risk estimates. The novel inclusion of HAP provides a more complete representation of the global burden attributable to PM_2.5_.
- This study provides separate burden estimates for ambient and household PM_2.5_ and describes uncertainty in the estimated attributable burden. It also includes an updated review of largest number of published studies to date, from all inhabited continents including high-pollution countries.

**What do these findings mean?:**

- LBW and PTB are key risk factors for early life mortality and lifetime morbidity. The impact of PM_2.5_ air pollution on these perinatal health outcomes contributes substantially to the overall global disease burden attributable to air pollution.
- This attributable burden assessment extends the impact of air pollution from diseases primarily impacting older adults.
- Implementation of air quality management and other approaches to reduce PM_2.5_ exposure may lead to large reductions in the global incidence of LBW and PTB infants and the associated disease burden. Mitigation measures even in low-exposure regions will likely manifest in significant improvement in these outcomes because the increase in risk is steeper at lower than in higher exposures, as shown by our risk curves.

## Introduction

The World Health Organization (WHO) estimated that 20 million infants were born low birth weight (LBW: birth weight less than 2,500 grams) and 15 million were preterm births (PTBs: gestation less than 37 completed weeks) in 2014 to 2015 [1]. The Global Burden of Disease (GBD) 2019 Study attributed 29% of the global under-5 mortality to short gestation and 34% to LBW [2]. Additionally, 13.2 million years lived with disability were attributed to PTB [3].

Several modifiable risk factors such as smoking, nutrition, and prepregnancy weight have been identified as risk factors for LBW, PTB, and reduction in birth weight and gestational age (GA) at birth [4,5]. Evidence for exposure to particulate matter <2.5 micrometer (PM_2.5_) in ambient air was classified as “suggestive of, but not sufficient to infer, a causal relationship” by the United States Environmental Protection Agency (USEPA) [6], with accumulating data from observational studies [7–13]. Exposure to PM_2.5_ from ambient and household air pollution (HAP) sources (such as use of solid fuels for cooking) is widespread and a major risk factor for global disease burden. For example, in 2019, 92% of the world’s population lived in areas that exceeded WHO guideline (annual average, 10 μg/m^3^) for PM_2.5_, and 3.8 billion people (49% of the global population) were exposed to HAP from the use of solid fuels for cooking [14]. Given this high exposure prevalence and growing epidemiologic literature on perinatal outcomes, there is a need to critically examine the evidence and assess the burden. Additionally, given that the majority of epidemiologic studies have been conducted in locations with lower pollution levels, disease burden and health impact assessments require an understanding of the shape of the risk relationship at levels encountered in high-pollution settings. With high PM_2.5_ exposure prevalence and a high incidence of LBW and PTB in many populations, such as in South Asia and sub-Saharan Africa, a small relative risk can yield a large attributable burden. An assessment of the global burden of these outcomes attributable to PM_2.5_ is therefore timely and necessary to provide evidence for policy action.

We conducted a systematic review (SR) and meta-regression (MR) following the Preferred Reporting Items for Systematic Reviews and Meta-Analyses (PRISMA) guidelines to quantify the relationships between average PM_2.5_ exposure during entire pregnancy and four adverse perinatal outcomes (reduction in birth weight and GA at birth, LBW, and PTB). Using the studies included in the SR–MR and a Bayesian regularized trimming method, an exposure–response curve was generated for each outcome, covering the global range of exposures, including those from HAP [15], with uncertainty intervals (UIs) accounting for between-study heterogeneity in risk estimates. Finally, we estimated the reduction in birth weight and GA at birth as well as the global proportion of LBW and PTB attributable to exposure to PM_2.5_ in ambient and household air. The article provides (1) methodologic description about the process of the novel inclusion of perinatal outcomes in GBD 2019 [2]; and (2) the first ever estimation, to our knowledge, of global burden of perinatal outcomes attributable to ambient, household, and total PM_2.5_ exposure during pregnancy for 204 countries and territories including all WHO member states.

## Methods

### Systematic review and meta-regression

We searched major databases for peer-reviewed articles in English that quantified the relationship of exposure to ambient and household PM_2.5_ pollution with four perinatal health indicators—birth weight (continuous), GA at birth (continuous), LBW (categorical), or PTB (categorical), published anytime until April 4, 2021 (Table A in S1 Text). Search strategy is presented in Figs A and B in S1 Text. We only found one estimate for continuous GA related to HAP [16]. Our study did not have a prespecified analysis plan. However, we used the standard methods for MR and for burden estimation as described below. For reporting, we used the PRISMA guidelines and the 2020 Checklist, which is presented in S1 PRISMA Checklist.

PM_2.5_ was selected as it is the most extensively studied pollutant in terms of impacts on perinatal outcomes in epidemiologic analyses. It has also been causally associated with several chronic diseases [17]. Further, global exposure models needed for burden assessment are available for PM_2.5_, and it is the primary metric used for assessment of burden attributable to ambient and HAP within the GBD. A global model for ozone and nitrogen dioxide is available, but few studies have examined these pollutants in relation to perinatal outcomes [18].

The inclusion criteria were cohort and case–control studies with medical subject headings—birth weight, LBW, PTB, GA, particulate air pollution, and PM_2.5_ for any calendar year, conducted on humans and investigated entire pregnancy exposure. Studies that reported any one or more of the four outcomes with PM_2.5_ exposure were included. Birth weight and LBW studies with or without restriction to term births were included. Studies were excluded if they were on animals, cigarette smoke, environmental tobacco smoke, secondhand smoke, or investigated short-term exposures. As our objective was to assess the risk from household and ubiquitous ambient exposure, occupational and accidental exposure studies were excluded. Occupational exposures are often order of magnitude higher than ambient and not experienced by the general population. We also excluded studies based on repeated pregnancies or multiple gestations (as they measured qualitatively different relationship) and those that pooled multiple cohorts if there was risk of double counting or overlap. If there were two articles on the same cohort, we included the study with the larger sample or that covered a longer period. The full list of articles was independently reviewed by RG and KC/SW. Differences were resolved by consensus in discussions between RG, KC, KB, SW, MB, and AC. The full list of studies including those that were excluded from the MR, with reasons for exclusion, are presented in S1 Table.

Two studies [19,20] reported nonlinear exposure–outcome relationship, which were included in the MR after converting to linear estimates. We used the area under the receiver operating characteristic (ROC) curve to reparametrize the nonlinear estimate to obtain the risk for the fifth to the 95th percentiles change in exposure. Using the magnitude of the nonlinear effect for the fifth to the 95th percentiles increment and assuming that the two points on the curve were connected by a straight line, we rescaled the effect size per linear 10 μg/m^3^ increment. The approximation helped minimize exclusion of important studies such as the one by Jedrychowski and colleagues, which was prospective, longitudinal, and used personal monitoring to assess exposure [19]. We included studies that reported results for PM_2.5_ categories, where it was possible to retrieve necessary information, using the method proposed by Hamling and colleagues [21], which assumes correlation between estimates for different exposure categories, to produce unbiased estimates.

We conducted MRs for the three outcomes with available studies, birth weight, LBW, and PTB. For birth weight and LBW, we estimated summary effects pooling all studies and separately for those that were restricted to term births only. There were insufficient studies reporting continuous GA for MR, but impacts on categorical PTB were transformed into estimated GA reductions for nonlinear risk curves, as described in more detail in the following section.

To evaluate residual confounding in individual studies, we examined several study characteristics including confounder adjustment. The variables considered for confounder adjustment were infant sex, socioeconomic status (SES), weight gain during pregnancy, exposure to tobacco smoke, and GA for birth weight and LBW. If the final models were adjusted for all of the above variables, we considered the study adjustment to be sufficient; otherwise, adjustment was considered insufficient. Confounding due to co-pollutant exposure was also considered, but the majority of the studies did not examine or report on co-pollutants. Likewise, residential mobility during pregnancy was seldom reported by the studies.

Study quality and risk of bias in individual studies were assessed using the Agency for Healthcare Research and Quality checklist [22] and the National Institute of Environmental Health Sciences (NIEHS) risk of bias questions [23]. Potential for bias in each study was assessed and identified as low, medium, high, or unclear. Specifically, we focused on four aspects that constitute major risk for bias in air pollution and perinatal outcomes studies: (1) exposure assessment [extrapolation from stationary monitors, spatiotemporal model, satellite aerosol optical depth (AOD) calibrated using ground-based monitor measurements and personal monitoring]; (2) residual confounding [adjustment for four variables were considered—GA (for birth weight or LBW), tobacco smoke, SES, and weight gain during pregnancy]; (3) confounding from co-pollutants (un)adjustment (i.e., percentage change in the PM_2.5_ association comparing single with two pollutant models); and (4) accounting for residential mobility during pregnancy in the exposure assessment (yes or no). Bias potential in a study was considered to be high if any two or more of the four above-stated criteria were present, medium if anyone was present, and low if none was present. In the MR, we quantitatively adjusted the summary estimate using our assessment of potential for bias in the individual studies.

Summary effects were generated using the restricted maximum likelihood method and reported per 10 μg/m^3^ increment in PM_2.5_. To examine the robustness of the summary effect in the MR, we individually adjusted for study size, design, location, method of exposure assessment, adjustment for confounders, and potential for bias. Categories of the three latter variables used for adjustment are presented in the preceding paragraph. Heterogeneity was assessed using the *I*^*2*^ statistic [24]. Publication bias was assessed using funnel plots and Egger test for asymmetry [25,26]. Our interpretations were not unduly based on *p*-values, rather they were more contextual, in line with recent recommendations from a large body of researchers [27].

### Risk curves (meta-regression–Bayesian regularized trimmed)

Using a novel tool, meta-regression–Bayesian regularized trimmed (MRBRT) [15], we created four nonlinear risk curves to estimate the risks of LBW and PTB and the shifts in birth weight (g) and GA (weeks) for a PM_2.5_ exposure distribution. These curves describe a summary risk and an UI of the relationships between PM_2.5_ exposure and each perinatal outcome. For the ambient studies included in the MR, we used the fifth and 95th percentiles from their PM_2.5_ distribution to estimate the corresponding relative risk. When these were not available, we used the mean and standard deviation, median and interquartile range (IQR), or minimum and maximum of exposure to estimate the fifth to 95th percentile and scaled the study estimates to these percentiles.

We defined HAP as the exposure to PM_2.5_ due to the use of solid fuels (dung, agricultural residues, wood, coal, and charcoal) for cooking, as in previous GBD studies [17,28]. Most of the HAP studies compared those using solid fuel for cooking to those who did not [except Wylie and colleagues who reported the change in birth weight (g) per IQR increase in measured PM_2.5_ [29]]. For studies reporting a binary (yes/no) HAP exposure, PM_2.5_ exposure was quantified using GBD methodology as described in Shupler and colleagues [30]. Briefly, exposure due to HAP is based on the relationships between HAP exposure and location, year, and subject (men, women, and children) measurements of PM_2.5_, which also accounts for the types of PM_2.5_ measurement (personal versus kitchen monitoring, duration of monitoring, etc.). Based on lag distributed income (LDI) per capita (a measure of development) of a given location and year, we estimated the excess HAP exposure after subtracting the year and location-specific ambient levels. The relative risk (RR) or beta coefficient of the HAP studies represents the change in risk between the estimated ambient level of exposure (Z_CF_) and the sum of the ambient level and the excess HAP exposure (Z) for a given study location and year.

Ambient PM_2.5_ was estimated from multiple satellite retrievals of AOD, a chemical transport model to relate column measurements of AOD to surface PM_2.5_ concentrations, and calibrated to available ground monitor measurements of PM_2.5_. These inputs were combined in a spatiotemporal Bayesian hierarchical model as described in detail previously [31].

Using the GBD 2019 predicted joint distributions of birth weight and GA in a study’s location and year [17], we transformed studies measuring LBW and PTB categorically into continuous shifts in birth weight (grams) and GA (weeks), respectively. In this way, we were able to use both categorical and continuous studies in the birth weight and GA risk curves. We tested various model settings and priors. The MRBRT models used third-order splines with three interior knots and a constraint on the right-most segment, forcing the fit to be linear rather than cubic. We used an ensemble approach to knot placement, wherein 50 different models were run with randomly placed knots and then combined by weighting based on a measure of fit that penalizes excessive changes in the third derivative of the curve. Knots were free to be placed along the entire domain of the data. We included shape constraints so that the risk curves were concave downwards and monotonically increasing for LBW and PTB and concave upwards and monotonically decreasing for birth weight and GA, the most biologically plausible shapes for the PM_2.5_ risk curve. On the nonlinear segments, we included a Gaussian prior on the third derivative of mean 0 and variance 1^−4^ to prevent overfitting; on the linear segment, a stronger prior of mean 0 and variance 1^−6^ was used to ensure that the risk curves do not continue to increase beyond the range of the exposure. We fit the splines on the following formulas:

For LBW and PTB (categorical):

$$log\left(\frac{MRBRT(Z)}{MRBRT(Z_{CF})}\right)∼log(Published\;Effect\;Size)$$

And for birth weight and GA (continuous):

$$MRBRT(Z)-MRBRT(Z_{CF})∼Published\;Shift$$

The same set of studies included in the SR–MR were used to generate the MRBRT risk curves for the four outcomes (Fig Ca–Cd in S1 Text). The horizontal lines represent the fifth and 95th percentiles of the PM_2.5_ exposure range for each of the individual epidemiologic studies. The MRBRT risk curves are conservative because of the Bayesian framework. Strong priors have been imposed on the curves so that at the higher end of the spline pertaining to high exposures, the risk is approximately flat. There were little data from available evidence to suggest further increases in risk above these levels.

To generate 95% UI, 1,000 exposures were predicted across the range of the curves. We incorporated predictions of between-study heterogeneity using the Fisher scoring correction to the heterogeneity parameter when creating these draws. To propagate uncertainty in the risk curves to the estimation of attributable burden, 1,000 risk estimates were generated for each exposure ranging from 0 to 2,500 μg/m^3^. In this analysis, we used a uniform counterfactual distribution from 2.4 to 5.9 μg/m^3^ as theoretical minimum risk exposure levels (TMRELs) [17]. TMREL is a uniform distribution with upper and lower bounds obtained from the average of the minimums and the fifth percentiles of ambient air pollution cohort studies conducted in North America. TMREL was chosen as a distribution rather than a fixed value to represent the uncertainty of the level of exposure consistent with the null effect [17]. The RRs used for burden analysis for categorical LBW and PTB outcomes took the following form:

$$for\;X\leq X_{CF},{\cdots \cdots \cdots \cdots RR}_{oap}(x)=1,\;RR_{hap}(x)=1$$ (1)

$$for\;ambient\;pollution\;X>X_{CF},{\cdots \cdots \cdots \cdots RR}_{oap}(x)=\frac{MRBRT(X_{oap})}{MRBRT(TMREL)}$$ (1A)

$$for\;household\;pollution\;X>X_{CF},{\cdots \cdots \cdots \cdots RR}_{hap}(x)=\frac{MRBRT(X_{oap+hap})}{MRBRT(TMREL)}$$ (1B)

where X is the value of PM_2.5_, X_CF_ is the TMREL, and MRBRT (X) is the RR for the value of X, and MRBRT (TMREL) is the RR for the value of X_CF_ from the MRBRT risk curve. The subscripts oap refers to ambient and hap refers to HAP, respectively. The beta coefficients for continuous birth weight and GA took the following form:

$$for\;X\leq X_{CF},{\cdots \cdots \cdots \cdots \beta }_{oap}(x)=0,\;\beta_{hap}(x)=0$$

$$for\;ambient\;pollution,X>X_{CF},{\cdots \cdots \cdots \cdots \beta }_{oap}(x)=MRBRT(X_{oap})-MRBRT(TMREL)$$ (2A)

$$for\;household\;pollution,X>X_{CF},{\cdots \cdots \cdots \cdots \beta }_{hap}(x)=MRBRT(X_{oap+hap})-MRBRT(TMREL)$$ (2B)

Supported by the SR–MR, we made several assumptions for the MRBRT curves, which are: (1) exposure to ambient and household PM_2.5_ reduces birth weight and GA and increases the risk of LBW and PTB; (2) the observed effects are functions of PM_2.5_ mass concentrations; and (3) the increased risk is based on long-term exposure, i.e., over the entire pregnancy. Additionally, we also assumed that the exposure–outcome relationships are not necessarily linear over the range of nonoccupational and nonaccidental human exposures.

### Estimation of global burden

We estimated the global and country-specific reductions in continuous birth weight and GA as well as the population attributable fractions (PAFs) and the incident cases (population attributable numbers [PANs]) for LBW and PTB. We used country-specific total live birth counts, LBW and PTB proportions, and annual PM_2.5_ exposures used for GBD 2019 [2,31]. The burden estimation used the risks from an updated MRBRT that included studies up to April 2021. The PAFs were estimated using the risks from MRBRT that cover a wide exposure range including both ambient and household sources. The specific steps are described below.

Step 1: A total of 1,000 simulated draws of outcome-specific RRs from the MRBRT risk curve for an exposure were matched with the annual average PM_2.5_ exposures for each country.
Step 2: A total of 1,000 draws of outcome-specific RRs corresponding with 1,000 TMREL values (ranging from 2.4 to 5.9 μg/m^3^) were merged with the 1,000 simulated draws of PM_2.5_ exposure for each country, so that draw 1 of exposure from step 1 corresponded with draw 1 of TMREL for a country. The loop was repeated for all 204 countries and territories.
Step 3: Next, the RRs and the beta coefficients were adjusted using the risks for the TMREL values, as shown in Eqs 1A, 1B, 2A, and 2B.
Step 4: The RR_pm_ and β_pm_ for total PM_2.5_ exposure were estimated as shown in Eqs 3A and 3B, where the ambient and HAP-specific RRs and βs are obtained from Eqs 1 and 2 above, respectively, and Prev_hap_ is the countrywide average prevalence of HAP for 2019 for females.

$${RR}_{pm}={RR}_{oap}(1-{Prev}_{hap})+{RR}_{hap}\times {Prev}_{hap}$$ (3A)

$$\beta_{pm}=\beta_{oap}(1-{Prev}_{hap})+\beta_{hap}\times {Prev}_{hap}$$ (3B)

Step 5: The 1,000 RR_pm_ generated in Eq 3A for total PM_2.5_ were used to generate 1,000 PAFs for each country (i) using the Eq 4. The mean of the 1,000 PAFs generated the country-specific ${PAF}_{{pm}_{i}}$along with the 95% UI.

$${PAF}_{{pm}_{i}}=\frac{{RR}_{{pm}_{i}}-1}{{RR}_{{pm}_{i}}}$$ (4)

Step 6: The global PAF was generated by weighting the country-specific ${PAF}_{{pm}_{i}}$ with the country-specific livebirth counts (Livebirth_i_) for the year 2019, as shown in Eq 5A. Similarly, the global reduction in birth weight and GA was generated by weighting the country-specific reductions with the corresponding 2019 livebirth counts, as shown in Eq 5B.

$$\frac{\sum_{i=1}^{204}{(PAF}_{{pm}_{i}}\times {Livebirth}_{i})}{\sum_{i=1}^{204}{Livebirth}_{i}}$$ (5A)

$$\frac{\sum_{i=1}^{204}{(\beta }_{{pm}_{i}}\times {Livebirth}_{i})}{\sum_{i=1}^{204}{Livebirth}_{i}}$$ (5B)

Step 7: The total PAF was apportioned in to PAF_oap_ and PAF_hap_ using Eqs 6A and 6B below. For birth weight and GA, we used Eqs 6C and 6D.

$${PAF}_{{oap}_{i}}=\frac{X_{{oap}_{i}}}{X_{{oap}_{i}}+{(Prev}_{{hap}_{i}}\times X_{{hap}_{i}})}\times {PAF}_{{pm}_{i}}$$ (6A)

$${PAF}_{{hap}_{i}}=\frac{{Prev}_{{hap}_{i}}\times X_{{hap}_{i}}}{X_{{oap}_{i}}+{(Prev}_{{hap}_{i}}\times X_{{hap}_{i}})}\times {PAF}_{{pm}_{i}}$$ (6B)

$$\beta_{{oap}_{i}}=\frac{X_{{oap}_{i}}}{X_{{oap}_{i}}+X_{{hap}_{i}}}\times \beta_{{pm}_{i}}$$ (6C)

$$\beta_{{hap}_{i}}=\frac{X_{{hap}_{i}}}{X_{{oap}_{i}}+X_{{hap}_{i}}}\times \beta_{{pm}_{i}}$$ (6D)

Step 8: The PANs were estimated using the ${PAF}_{{pm}_{i}}$, the proportions of LBW_i_ or PTB_i_, and the livebirths (Livebirth_i_), all at the country level, for 2019 as shown in Eq 7. The global PANs for LBW and PTB were estimated by adding the country-specific PANs. The estimated country-level burden can be interpreted as the increase in the mean (birth weight and GA) or decrease in the incident cases (LBW and PTB) if the exposures were reduced to the TMREL. Analysis was conducted in STATA MP Version 17 (StataCorp, College Station, Texas, USA).

$${PAN}_{{pm}_{i}}={PAF}_{{pm}_{i}}\times ({LBW}_{i}\;or\;{PTB}_{i})\times {Livebirth}_{i}$$ (7)

## Results

### Systematic review and meta-regression

The MR included 44 studies on birth weight, 40 studies on LBW, and 40 studies on PTB that investigated association with ambient PM_2.5_. Figs A and B in S1 Text sequentially present the results of keyword search to the final selection of studies, and the reasons for exclusions are described in S1 Table. All the studies were observational with majority of retrospective cohort design and primarily from North America, Europe, and Australia. A few recent studies were from China, India, sub-Saharan Africa, and South America (S1 Table).

The summary linear estimate from the 44 studies shows −22 grams (95% UI: −32, −12) lower birth weight per 10 μg/m^3^ increase in the entire pregnancy average PM_2.5_ exposure (Table 1). The estimate changes to −35 (95% UI: −55, −15) when restricted to 13 studies that included all births (i.e., these studies did not exclude PTB). Adjustment for the methods adopted by the individual studies to assess exposure and potential for bias in the individual studies changed the associations to −12 grams (95% UI: −52, 27) and −1 gram (95% UI: −62, 60), respectively. There was no evidence of any study having excessive influence on the summary estimate (Fig Da in S1 Text). However, the between-study heterogeneity (*I*^*2*^) was >99%. The funnel plot shows that studies were relatively evenly distributed on both sides of the null value (Fig Ea in S1 Text), and the Egger test (*p* = 0.34) was nonsignificant.

**Table 1:** Results after adjusting the summary effect with study characteristics and sources of heterogeneity.

	Summary effect (95% confidence interval)	*p*-value
Birth weight^1^ (*n* = 44)^2^		
**Summary effect**	**−22.4 (−32.4, −12.3)**	**<0.001**
Summary effect + study size	**−**23.3 (**−**34.4, **−**12.2)	<0.001
Summary effect + study region	**−**23.0 (**−**34.5, **−**11.4)	<0.001
Summary effect + study design	**−**24.3 (**−**35.6, **−**13.0)	<0.001
Summary effect + exposure assessment method	**−**12.1 (**−**51.7, 27.4)	0.547
Summary effect + level of confounder adjustment	**−**26.7 (**−**40.1, **−**13.3)	<0.001
Summary effect + potential for bias in a study	**−**0.9 (**−**61.9, 60.0)	0.976
Low birth weight^3^ (*n* = 40)		
**Summary effect**	**1.11 (1.07, 1.16)**	**<0.001**
Summary effect + study size	1.15 (1.09, 1.21)	<0.001
Summary effect + study region	1.07 (1.02, 1.13)	0.004
Summary effect + study design	1.10 (1.05, 1.16)	<0.001
Summary effect + exposure assessment method	1.02 (0.81, 1.29)	0.844
Summary effect + level of confounder adjustment	1.16 (1.07, 1.26)	0.001
Summary effect + potential for bias in a study	1.22 (1.02, 1.45)	0.027
Preterm birth^3^ (*n* = 40)		
**Summary effect**	**1.12 (1.06, 1.19)**	**<0.001**
Summary effect + study size	1.14 (1.06, 1.23)	<0.001
Summary effect + study region	1.11 (1.02, 1.20)	0.012
Summary effect + study design	1.11 (1.05, 1.18)	0.001
Summary effect + exposure assessment method	1.07 (0.93, 1.23)	0.366
Summary effect + level of confounder adjustment	1.16 (1.06, 1.27)	0.001
Summary effect + potential for bias in a study	1.25 (1.00, 1.57)	0.053

^1^ Decrease in birth weight (grams), beta coefficient.

^2^ “*n*” is the number of studies included in the meta-regression.

^3^ Elevated risk for the outcome.

The estimates are beta coefficients or risks per 10 μg/m^3^ increase in the entire pregnancy average ambient particulate matter <2.5 micrometer exposure.

The adjustment variables are described in the Methods section and presented in the study characteristics Supporting information table.

The summary linear estimate for LBW was 11% greater risk (1.11 95% UI: 1.07, 1.16) per 10 μg/m^3^ increase in entire pregnancy average PM_2.5_ exposure (Fig Db in S1 Text). The estimate changed to 1.25 (95% UI: 1.06, 1.48) when restricted to 9 studies that included all births. Adjustment for methods of exposure assessment and potential for bias in the individual studies changed the summary estimate to 1.02 (95% UI: 0.81, 1.29) and 1.22 (95% UI: 1.02, 1.45), respectively (Table 1). Other adjustments did not change the summary estimate substantially, neither was there any evidence of a study having excessive influence (Fig Db in S1 Text). Between-study heterogeneity (*I*^*2*^) was 95%, the funnel plot shows evidence of asymmetry (Fig Eb in S1 Text), and the Egger test (*p* < 0.001) was significant.

The summary linear estimate for PTB was 12% greater risk (1.12 95% UI: 1.06, 1.19) per 10 μg/m^3^ increase in entire pregnancy average PM_2.5_ exposure (Fig Dc in S1 Text). Adjustment for methods of exposure assessment and potential for bias in the individual studies changed the summary estimate to 1.07 (95% UI: 0.93, 1.23) and 1.25 (95% UI: 1.00, 1.57), respectively. Adjustment for exposure assessment method attenuated the summary estimate, which became nonsignificant (Table 1). There was no evidence of excessive influence of any study on the summary estimate (Fig Dc in S1 Text). The between-study heterogeneity (*I*^*2*^) was >99%. The funnel plot shows evidence of asymmetry; smaller studies tended to show positive effect, while relatively larger studies were evenly distributed on both sides of the null value (Fig Ec in S1 Text). The Egger test (*p* = 0.93) was nonsignificant.

### Global exposure levels

**Fig 1:**
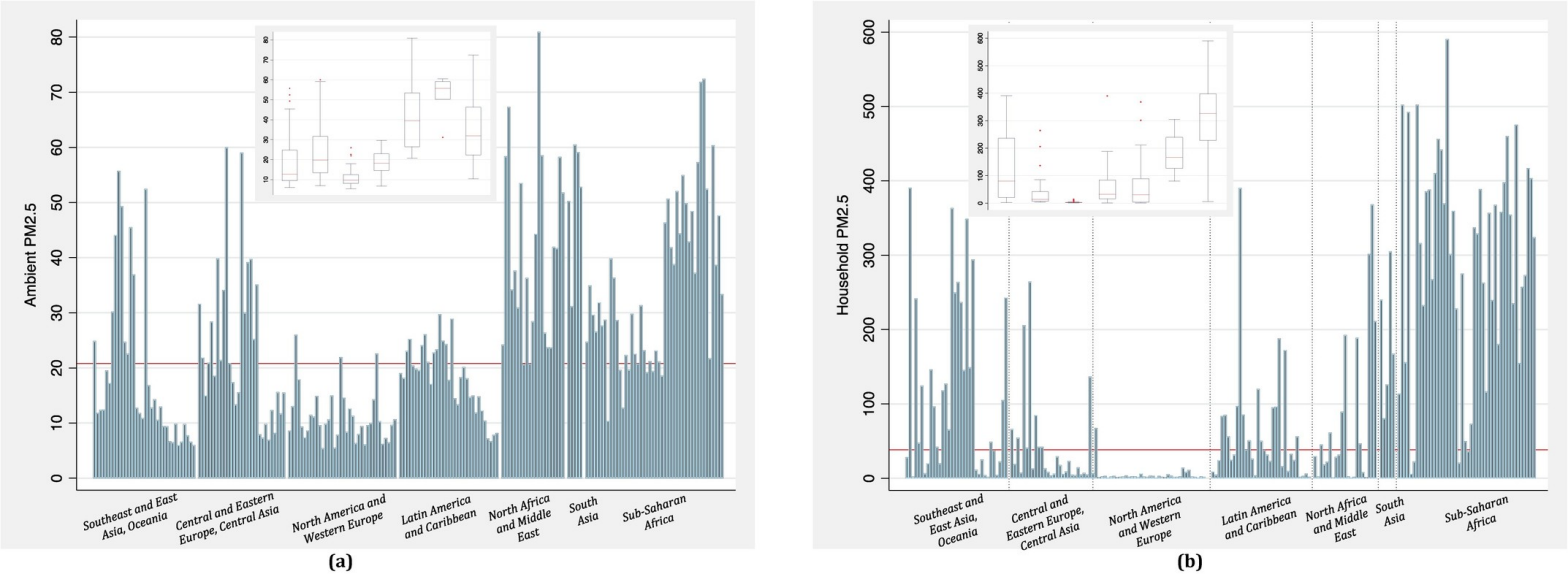
The annual average ambient (a) and household (b) PM_2.5_ concentrations (μg/m^3^) in 204 countries and territories for 2019. The box plots in the inset show the super regional distributions. Note: The red horizontal line in the overall plots represent the global median, and those within the boxes are the GBD 2019 super regional medians. The boxplots are arranged in the same order as the super regions in the overall plot. GBD, Global Burden of Disease, Injuries, and Risk Factors; PM_2.5_, particulate matter <2.5 micrometer.

The global medians and the IQRs for ambient and HAP PM_2.5_ were 20.8 (11.7 to 33.7) and 38.4 (5.2 to 208.6) μg/m^3^, respectively, for 2019 (Fig 1). The median ambient PM_2.5_ levels by the 7 GBD super regions was the lowest in North America and Western Europe (9.8 μg/m^3^) and the highest in South Asia (55.7 μg/m^3^). Likewise, median HAP PM_2.5_ levels by the 7 super regions was the lowest in North America and Western Europe (2.3 μg/m^3^) and highest in sub-Saharan Africa (326.3 μg/m^3^).

### Global burden

**Fig 2:**
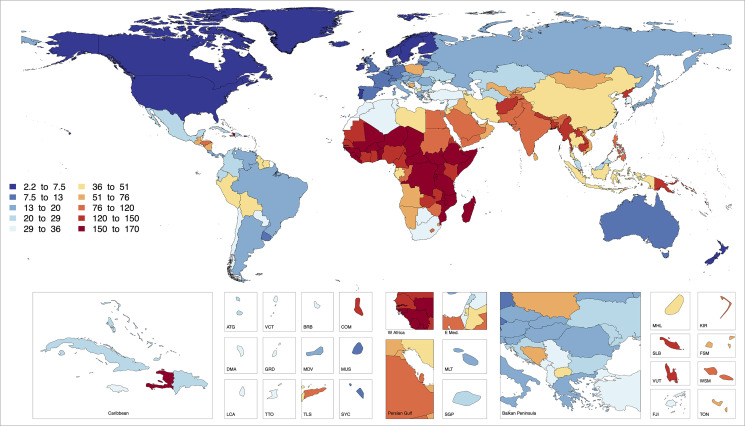
The estimated global reduction in population-weighted birth weight (grams) attributable to total PM_2.5_ air pollution (from ambient and household sources) for 2019. PM_2.5_, particulate matter <2.5 micrometer.

**Fig 3:**
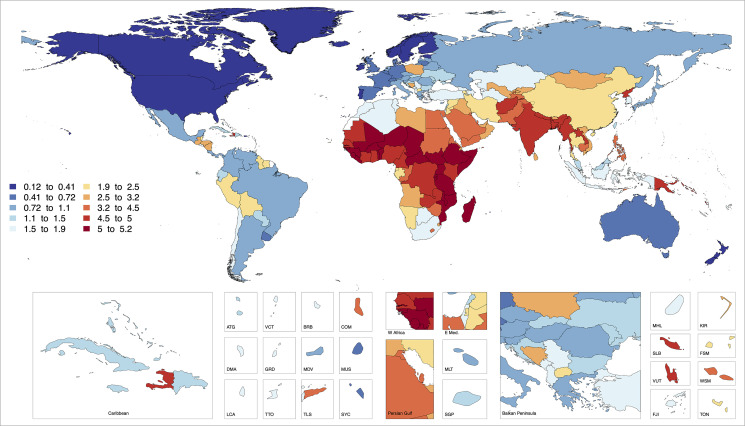
The estimated global reduction in population-weighted gestational age (weeks) attributable to total PM_2.5_ air pollution (from ambient and household sources) for 2019. The mapping function or the base layers for Figs 2 and 3 were obtained from this source: http://www.fao.org/geonetwork/srv/en/metadata.show?id=12691. PM_2.5_, particulate matter <2.5 micrometer.

The global population–weighted mean lowering of estimated birth weight in 2019 attributable to the total ambient and household PM_2.5_ exposure was 89 grams (95% UI: 88, 89) (Fig 2). In other words, population-weighted mean birth weight would have been 89 grams higher if the exposures were at the TMREL. Regionally, the highest reductions were estimated for South Asia (118 grams) and sub-Saharan Africa (140 grams), while the lowest were in North America and Western Europe (11 grams), with country-specific reductions ranging from 2 grams (95% UI: 2, 2) in Finland to 161 grams (95% UI: 161, 161) in Central African Republic. The global population–weighted mean lowering of estimated GA at birth attributable to total PM_2.5_ was 3.4 weeks (95% UI: 3.4, 3.4), with trends across GBD regions and countries similar to those for birth weight (Fig 3). Of the total attributable global reductions in birth weight and GA at birth, about one quarter was due to ambient and three quarters due to HAP PM_2.5_ exposure. Country-specific reductions are presented in Figs 2 and 3 and in S2 Table.

**Fig 4:**
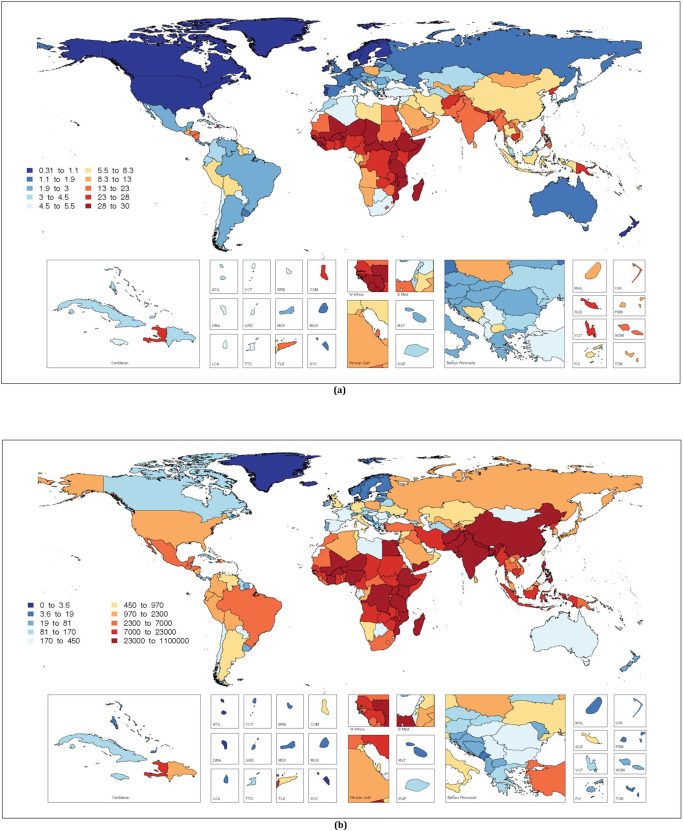
The estimated global burden [PAFs (a) and PANs (b)] of low birth weight attributable to total PM_2.5_ air pollution (from ambient and household sources) for 2019. PAF, population attributable fraction; PAN, population attributable number; PM_2.5_, particulate matter <2.5 micrometer.

**Fig 5:**
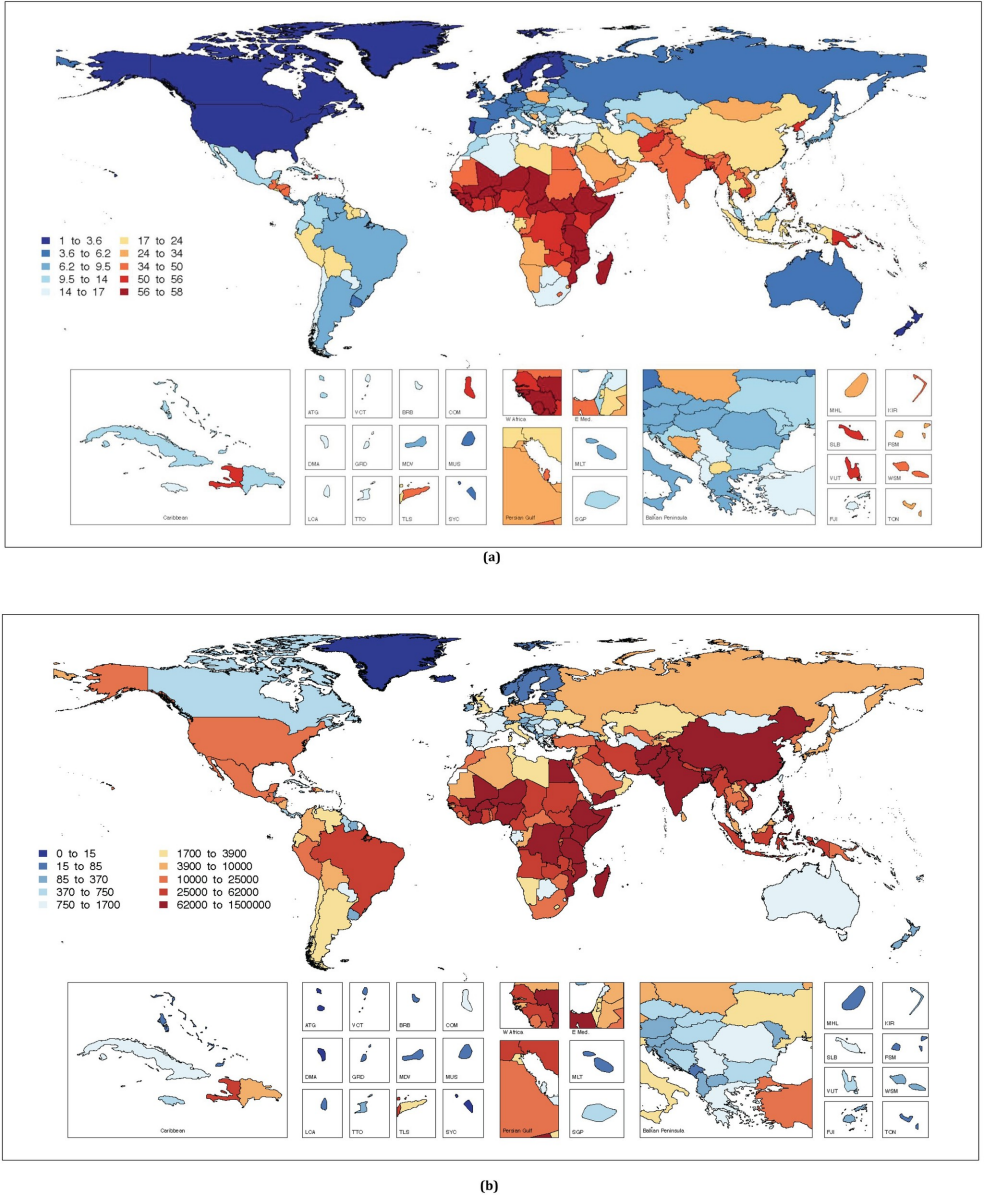
The estimated global burden [PAFs (a) and PANs (b)] of preterm birth attributable to total PM_2.5_ air pollution (from ambient and household sources) for 2019. The mapping function or the base layers for Figs 4 and 5 were obtained from this source: https://data.apps.fao.org/map/catalog/srv/eng/catalog.search#/metadata/9c35ba10-5649-41c8-bdfc-eb78e9e65654. PAF, population attributable fraction; PAN, population attributable number; PM_2.5_, particulate matter <2.5 micrometer.

An estimated 15.6% (95% UI: 15.6, 15.7) of all LBW infants globally could be attributed to exposure to total PM_2.5_, i.e., 2,761,720 LBW infants (95% UI: 2,746,713 to 2,776,722), for the year 2019 (Fig 4A and 4B). The burden was the highest in South Asia (20.9%) and lowest in North America and Western Europe (4.9%), while the country-specific attributable burdens range from 0.3% in Finland (95% UI: 0.3, 0.3) to 29.3% (95% UI: 29.2, 29.3) in Central African Republic. In 2019, 35.7% (95% UI: 35.6, 35.9) of all PTB infants globally could be attributed to total PM_2.5_ exposure, accounting for 5,870,103 (95% UI: 5,848,046 to 5,892,166) PTB infants (Fig 5A and 5B). The highest attributable burden for PTB was estimated for sub-Saharan Africa (52.5%), and the country-specific estimates ranged from 1.1% in Finland (95% UI: 1.0, 1.1) to 57.2% in Central African Republic (95% CI: 57.2, 57.3). A little over one-third of the total PM_2.5_ burden for LBW and PTB was due to ambient PM_2.5_. Ambient- and HAP-specific reductions in birth weight and GA at birth as well as PAFs and PANs for LBW and PTB for each country are presented in S2 Table.

## Discussion

To our knowledge, this is the first assessment of global burden of adverse perinatal outcomes that includes both ambient and HAP. Our findings suggest that about 2.8 million LBW and 5.9 million PTB infants, globally, could have been averted in 2019 if the mean PM_2.5_ exposure during the entire pregnancy was reduced to the TMREL. South Asia and sub-Saharan Africa combined could have decreased the 2019 LBW and PTB incidence by about 78%. LBW and PTB are leading contributors toward neonatal mortality, and these two regions register high incidence of the two outcomes. Globally, modest decreases in birth weight and relatively large decreases in GA at birth were attributable to long-term PM_2.5_ exposure during pregnancy. South Asia and sub-Saharan Africa registered the highest decreases in birth weight and GA and, in these regions, population level mean birth weight and GA are lower than in high-income countries. The results indicate that PM_2.5_ induced a modest shift in the mean of the distributions of birth weight and GA, which, in turn, could be responsible for a considerable part of the burden for LBW and PTB.

An assessment from the United States reported a burden of 15,808 PTBs (with a wide UI of 7,532 to 29,968) attributable to ambient PM_2.5_ in 2010, compared to our estimate of 11,646 (95% UI: 11,416 to 11,877) from ambient PM_2.5_ in 2019 [32]. Compared to the large uncertainty range of the prior estimate, differences between the 2 estimates could be likely due to two major factors—changes in the PM_2.5_ attributable burden in the previous decade and different input data as well as the methodologies used to estimate attributable burden. The different risk relationships, in particular, are likely important, especially the use of linear versus nonlinear relationships, given that the total number of births (approximately 3.9 million) and the proportion of PTB (approximately 12%) were similar in the two assessments. PM_2.5_ attributable burden has likely reduced since population-weighted average pollution levels in the US declined from 9.2 μg/m^3^ in 2010 to 7.7 μg/m^3^ in 2019 [2]. Compared to our global ambient estimate of 2.0 million PTB in 2019, Malley and colleagues [59] reported a global burden of 3.5 million PTB attributable to ambient PM_2.5_ in 2010. This difference very likely results from the extrapolation of a linear estimate generated by Sun and colleagues [10], who used only ambient air pollution studies, to the higher exposures experienced in middle- and low-income countries. In contrast, our nonlinear risk curves included both ambient and HAP studies, as well as more recent studies including several from high-pollution countries. Our curves indicate a reduced slope at higher exposures, suggesting that extrapolations of linear relationships will overestimate burden. Likewise, another study from Shanghai, China reported 23% and 33% PAF for LBW and PTB, respectively, in 2013 [33]. The study used a linear relationship with ambient PM_2.5_ for China reported by Fleisher and colleagues [34] and used a counterfactual of 15 μg/m^3^. In comparison, we estimated the 2019 ambient PM_2.5_ attributable burden for China to be 4.9% for LBW and 14.6% for PTB. Our development of nonlinear MRBRT risk curve is a substantial improvement over prior linear estimates, especially when applied across the full range of global exposures. Furthermore, the MRBRT tool allows for consideration of multiple study-level variables to address between-study heterogeneity in effect estimates and UIs, and our analysis included full uncertainty distributions.

This SR–MR included the largest number of studies to date, covering the global range of exposure and provides evidence for a quantitative relationship that is compatible with an adverse effect of PM_2.5_ exposure on perinatal outcomes. While heterogeneity between studies was present, it was expected given the diversities in population, size, design, exposure assessment, and covariate adjustment. In fact, considering the ubiquitous nature of PM_2.5_ pollution and potentially different risks in different populations, diversity between studies is a rare strength of this SR–MR as the evidence pool covers populations with different genetic makeup, SES strata, varying underlying physical makeup (e.g., short stature in Asian populations, high prevalence of malnutrition, etc.), different medical conditions, and air pollution exposure levels. PM_2.5_ remained associated with all outcomes in the MR that addressed multiple sources of bias. Evaluation of study level factors indicated that adjustment for exposure assessment methods attenuated the risk magnitude, suggesting that exposure misclassification was present in varying degrees and likely nondifferentially distributed across studies. This attenuation may also have to do with the accuracy of GA assessments, which, in turn, determines exposure duration. Adjusting for potential for bias in the individual studies led to complete attenuation of the risk for birth weight, while the risks for LBW and PTB almost doubled. There is no straightforward explanation for this finding, although it may reflect random variation in effect sizes across the different studies.

Our SR–MR results [an estimated 22 grams (95% UI: −32, −12) of lower birth weight and 11% (1.11 95% UI: 1.07, 1.16) and 12% (1.12 95% UI: 1.06, 1.19) greater estimated risks of LBW and PTB, respectively, per 10 μg/m^3^] are generally consistent with prior meta-analyses because all of these were quantified on a linear scale. The assumption of linearity is likely an oversimplification of the actual form of the relationships. The first meta-analysis (MA) of 20 studies published by Sapkota and colleagues in 2010 indicated summary estimates (odds ratio [OR]) of 1.09 for LBW and 1.15 for PTB, per 10 μg/m^3^ increment [7]. The next MA of 62 studies reported −23.2 grams reduction in birth weight and ORs of 1.05 for LBW and 1.05 for PTB, per 10 μg/m^3^ increment [9]. Additional reviews and meta-analyses indicated risks ranging from 13.9 to 22.2 grams reductions in birth weight and ORs of 1.09 to 1.10 and 1.03 to 1.15 for LBW and PTB, respectively, per 10 μg/m^3^ increment [8,10–13,35,36]. The two natural experiment studies also provide important evidence consistent with adverse effects of gestational PM_2.5_ exposure [37,38]. The Beijing study reported 23 grams more birth weight comparing pregnancies who had their eighth month during the 2008 Olympics Games with those who had their eighth month on the same time in 2007 and 2009 [38]. The Utah study reported lower risk of PTB among women who were pregnant during the local steel mill closure compared to women who were pregnant before or after closure [37].

Although our results are consistent with previous MAs and natural experiments, the summary relative effects were small, and the possibility for alternative explanations remains. We examined the extent of confounder adjustment in the individual studies and categorized them as sufficient or insufficient, as described in the Methods. Accounting for the extent of confounder adjustment increased the summary estimates for all three outcomes, suggesting that residual confounding is a potential source of bias and that risks in the original studies were likely to have been underestimated. GA is also considered to be on the causal pathway between PM_2.5_ exposure and reduced birth weight or LBW, and if there are unmeasured confounders, adjustment for GA likely biases the direct effect [39]. Consistent with the aim of quantifying the direct effect of PM_2.5_ on birthweight, the majority of the studies in the SR–MR either adjusted for GA or restricted the study population to term births, and, sometimes, both, inadvertently biasing the estimate. If we restrict the SR–MR to studies that did not account for GA, in one way or the other, the vast majority of the studies would be excluded. We have shown that the summary effects were higher for birth weight and LBW when using only those studies that included all births (i.e., did not exclude PTBs), compared to the estimate obtained from all eligible studies. Thus, adjustment for GA attenuated the risk, empirically confirming the concept alluded to by Wilcox and colleagues [39]. Since the risk relationships used in the burden assessment for birth weight or LBW were based on the same studies as the SR–MR, the global burden may therefore have been underestimated. Further, this underestimation likely affects only the LBW burden because the PTB burden is unlikely to be biased, as suggested by Wilcox and colleagues [39].

Studies of the effects of air pollution on fetal and maternal physiology provide additional evidence consistent with the mechanisms thought to contribute to adverse perinatal outcomes. Air pollution affects both the anatomy and the physiology of the placenta and the umbilical cord. Particles induce antiangiogenic profiles, leading to thinner and less voluminous umbilical cord affecting oxygen diffusion in murine models [40,41] and replicated in humans [42]. A human study has also shown that particles affect umbilical–placental circulation increasing blood flow resistance [43]. Air pollution induces hypoxemia, impairs trophoblast invasion and vascularization leading to uteroplacental hypoperfusion, thereby causing retarded fetal growth [44–46]. Particles species (e.g., PAH, B[a]P) bind with hydrocarbon receptors, causing mutagenesis and disrupting the human endocrine system [47]. Epidemiological studies have suggested that hydrocarbons form PAH-DNA adducts, activating apoptotic pathways, decreasing exchange through the placenta [48,49]. An in vitro study using human cells have shown particles can penetrate the placental barriers mediated by macrophages and the dendritic cells [50]. Engulfed particles are released into the blood stream triggering release of inflammatory mediators like cytokines, C-reactive proteins, and interleukins promoting systemic inflammation [51–55]. Inflammation is also caused by reactive oxygen and nitrogen species in particulates inducing oxidative stress compromising host defense, increasing vulnerability to maternal infections and premature contractions, and/or rupture of membranes [56], which are underlying causes for PTB. Particles have been associated with pregnancy-induced hypertensive disorders [57], increasing the risk of growth retardation and PTB either as a consequence of preexisting or pregnancy-induced hypertension [54,58].

### Limitations

The SR–MR was restricted to articles in English, as it was beyond the scope to include articles in other languages. We quantified the risk of exposure for the entire duration of pregnancy because the largest number of studies investigated this exposure window. However, other short- and long-term exposure windows may also be critical. We assumed no interaction between ambient and HAP exposure, consistent with the design of the epidemiologic analyses that contribute to the SR–MR. To our knowledge, we have not seen evidence to the contrary for any of the four outcomes investigated. If there is synergism between the two sources of PM_2.5_ and/or other factors (e.g., maternal weight gain during pregnancy), the real burden could be much higher, especially in low- and middle-income countries. As highlighted in the methods, we focused on PM_2.5_ to be consistent with the GBD analyses and because it is one of the most potent and extensively investigated pollutant measures in relation to these outcomes. As evidence accrues, additional pollutants including speciated or source-specific PM_2.5_ could be considered in the global burden estimation. Another limitation is the uncertainty in the assessment of ambient and HAP exposure. For example, we modeled HAP exposure based on a global database of short-term measurement studies, which are surrogates for exposures throughout the period of pregnancy. Further, the burden estimation incorporates the assumption that the mean population-level entire pregnancy average exposure was approximated by the country-level annual population-weighted average. We also assumed that the exposure and risk were constant over the duration of pregnancy. In other words, we did not have time-varying exposure over the duration of pregnancy to quantify varying risks over the course of the pregnancy. Our uncertainty distribution (1,000 risks for each outcome corresponding to 1,000 different exposures) likely accounted for some degree of the time-varying exposure that each individual may encounter. Finally, the burden estimates for LBW and PTB should not be interpreted as mutually exclusive because some of the LBW infants are also likely to be PTB.

### Divergence from GBD 2019 methods

In this paper, we have evaluated birth weight, GA, LBW, and PTB as outcomes and provide direct estimates of the burden of these outcomes that is attributable to PM_2.5_. This differs from the mediation of the burden of disease attributable to PM_2.5_ via short gestation and reduced birth weight that was first introduced in GBD 2019 using the methodology described here to estimate shifts in the distributions of birth weight and GA [2]. Specifically, the GBD estimated the impact of PM_2.5_ through shifts in birth weight and GA via a mediation analysis where LBW and PTB are risk factors for neonatal causes including mortality (due to diarrheal diseases, lower and upper respiratory infections, otitis media, meningitis, encephalitis, neonatal encephalopathy, neonatal sepsis, hemolytic disease, other neonatal jaundice, and other neonatal disorders) and years lived with disability attributable to PTB. Birth weight and GA were estimated with joint distributions with RR estimated for birth weight and GA categories. To do this, the MRBRT curves for birth weight and GA described here were used to shift the estimated birth weight and GA exposure distribution for a given location and year. This shifted distribution represented the expected distribution if PM_2.5_ was at the TMREL. By comparing the estimated to the expected (assuming exposure at the TMREL) distribution of birth weight and GA, we calculated PAFs for the stated outcomes to estimate mortality, years lived with disability, years of life lost, and disability-adjusted life years attributable to PM_2.5_ mediated through birth weight and GA. In the GBD 2019, 135,000 and 237,000 deaths from neonatal disorders were attributable to ambient and household PM_2.5_, respectively [2]. In addition, 326,000 and 423,000 deaths from lower respiratory infections were attributable to ambient and household PM_2.5_, respectively, a portion of which were mediated by reduced birth weight and short gestation.

This study investigated relevant indicators of perinatal health and provide strong evidence for PM_2.5_ exposure during pregnancy to be a risk factor for adverse outcomes across a wide range of exposures. We estimated that 2.8 million LBW and over 5.9 million PTB infants could be attributable to PM_2.5_ air pollution exposure during pregnancy in 2019. As these perinatal health indicators are key drivers of early life mortality, particularly in middle- and low-income countries, reducing air pollution will likely have substantial benefits for neonatal and infant health.

## Supporting information

S1 PRISMA Checklist PRISMA 2020 Checklist. PRISMA, Preferred Reporting Items for Systematic Reviews and Meta-Analyses. (DOCX)

S1 Text Supporting information methods for the SR and the MR. MR, meta-regression; SR, systematic review. (DOCX)

S1 Table Characteristics of the studies included in the MR. MR, meta-regression. (XLSX)

S2 Table Numerical estimates for the Figs 2–5 shown in each of the global maps. (XLSX)

## Abbreviations

AOD: aerosol optical depth
GA: gestational age
GBD: Global Burden of Disease
HAP: household air pollution
IQR: interquartile range
LBW: low birth weight
LDI: lag distributed income
MA: meta-analysis
MR: meta-regression
MRBRT: meta-regression–Bayesian regularized trimmed
NIEHS: National Institute of Environmental Health Sciences
OR: odds ratio
PAF: population attributable fraction
PAN: population attributable number
PM_2.5_: particulate matter <2.5 micrometer
PRISMA: Preferred Reporting Items for Systematic Reviews and Meta-Analyses
PTB: preterm birth
ROC: receiver operating characteristic
RR: relative risk
SES: socioeconomic status
SR: systematic review
TMREL: theoretical minimum risk exposure level
UI: uncertainty interval
USEPA: United States Environmental Protection Agency
WHO: World Health Organization

## References

[1] World Health Organization. Care of the preterm and low-birth-weight newborn. Geneva, Switzerland: WHO; 2019 [cited 2019 Oct 22]. Available from: https://www.who.int/maternal_child_adolescent/newborns/prematurity/en/.

[2] GBD 2019 Risk Factors Collaborators. Global burden of 87 risk factors in 204 countries and territories, 1990–2019: a systematic analysis for the Global Burden of Disease Study 2019. Lancet. 2020;396:1223–149. doi: 10.1016/S0140-6736(20)30752-2 33069327 PMC7566194

[3] James SL, Abate D, Abate KH, Abay SM, Abbafati C, Abbasi N, et al. Global, regional, and national incidence, prevalence, and years lived with disability for 354 diseases and injuries for 195 countries and territories, 1990–2017: a systematic analysis for the Global Burden of Disease Study 2017. Lancet. 2018;392(10159):1789–858. doi: 10.1016/S0140-6736(18)32279-7 30496104 PMC6227754

[4] Kramer MS. Determinants of low birth weight: methodological assessment and meta-analysis. Bull World Health Organ. 1987;65(5):663. 3322602 PMC2491072

[5] Berkowitz GS, Papiernik E. Epidemiology of preterm birth. Epidemiol Rev. 1993;15(2):414–43. doi: 10.1093/oxfordjournals.epirev.a036128 8174665

[6] United States Environment Protection Agency. Integrated science assessment for particulate matter. Washinton DC, USA: Environmental Protection Agency; 2018. 36630543

[7] Sapkota A, Chelikowsky AP, Nachman KE, Cohen AJ, Ritz B. Exposure to particulate matter and adverse birth outcomes: a comprehensive review and meta-analysis. Air Qual Atmos Health. 2012;5(4):369–81.

[8] Lamichhane DK, Leem J-H, Lee J-Y, Kim H-C. A meta-analysis of exposure to particulate matter and adverse birth outcomes. Environ Health Toxicol. 2015;30. doi: 10.5620/eht.e2015011 26796890 PMC4722965

[9] Stieb DM, Chen L, Eshoul M, Judek S. Ambient air pollution, birth weight and preterm birth: a systematic review and meta-analysis. Environ Res. 2012;117:100–11. doi: 10.1016/j.envres.2012.05.007 22726801

[10] Sun X, Luo X, Zhao C, Ng RWC, Lim CED, Zhang B, et al. The association between fine particulate matter exposure during pregnancy and preterm birth: a meta-analysis. BMC Pregnancy Childbirth. 2015;15(1):300. doi: 10.1186/s12884-015-0738-2 26581753 PMC4650291

[11] Zhu X, Liu Y, Chen Y, Yao C, Che Z, Cao J. Maternal exposure to fine particulate matter (PM 2.5) and pregnancy outcomes: a meta-analysis. Environ Sci Pollut Res Int. 2015;22(5):3383–96. doi: 10.1007/s11356-014-3458-7 25163563

[12] Sun X, Luo X, Zhao C, Zhang B, Tao J, Yang Z, et al. The associations between birth weight and exposure to fine particulate matter (PM2. 5) and its chemical constituents during pregnancy: A meta-analysis. Environ Pollut. 2016;211:38–47. doi: 10.1016/j.envpol.2015.12.022 26736054

[13] Li X, Huang S, Jiao A, Yang X, Yun J, Wang Y, et al. Association between ambient fine particulate matter and preterm birth or term low birth weight: an updated systematic review and meta-analysis. Environ Pollut. 2017;227:596–605. doi: 10.1016/j.envpol.2017.03.055 28457735

[14] Health Effects Institute. State of Global Air 2020 A Special Report. Boston, MA: Health Effects Institute; 2020.

[15] Zheng P, Aravkin AY, Barber R, Sorensen RJ, Murray CJ. Trimmed Constrained Mixed Effects Models: Formulations and Algorithms. arXiv. 2019.

[16] Alexander DA, Northcross A, Karrison T, Morhasson-Bello O, Wilson N, Atalabi OM, et al. Pregnancy outcomes and ethanol cook stove intervention: a randomized-controlled trial in Ibadan, Nigeria. Environ Int. 2018;111:152–63. doi: 10.1016/j.envint.2017.11.021 29216559

[17] GBD 2017 Risk Factor Collaborators. Global, regional, and national comparative risk assessment of 84 behavioural, environmental and occupational, and metabolic risks or clusters of risks for 195 countries and territories, 1990–2017: a systematic analysis for the Global Burden of Disease Study 2017. Lancet. 2018;392(10159):1923–94. doi: 10.1016/S0140-6736(18)32225-6 30496105 PMC6227755

[18] Larkin A, Geddes JA, Martin RV, Xiao Q, Liu Y, Marshall JD, et al. Global land use regression model for nitrogen dioxide air pollution. Environ Sci Technol. 2017;51(12):6957–64. doi: 10.1021/acs.est.7b01148 28520422 PMC5565206

[19] Jedrychowski W, Perera F, Mrozek-Budzyn D, Mroz E, Flak E, Spengler JD, et al. Gender differences in fetal growth of newborns exposed prenatally to airborne fine particulate matter. Environ Res. 2009;109(4):447–56. doi: 10.1016/j.envres.2009.01.009 19261271 PMC3786262

[20] Erickson AC, Ostry A, Chan LH, Arbour L. The reduction of birth weight by fine particulate matter and its modification by maternal and neighbourhood-level factors: a multilevel analysis in British Columbia, Canada. Environ Health. 2016;15(1):51. doi: 10.1186/s12940-016-0133-0 27079512 PMC4831087

[21] Hamling J, Lee P, Weitkunat R, Ambühl M. Facilitating meta-analyses by deriving relative effect and precision estimates for alternative comparisons from a set of estimates presented by exposure level or disease category. Stat Med. 2008;27(7):954–70. doi: 10.1002/sim.3013 17676579

[22] Viswanathan M, Berkman ND, Dryden DM, Hartling L. Assessing risk of bias and confounding in observational studies of interventions or exposures: Further Development of the RTI Item Bank. 2013. Report No.: 13-EHC106-EF.: . 24006553

[23] Rooney AA, Boyles AL, Wolfe MS, Bucher JR, Thayer KA. Systematic review and evidence integration for literature-based environmental health science assessments. Environ Health Perspect. 2014;122(7):711–8. doi: 10.1289/ehp.1307972 24755067 PMC4080517

[24] Higgins JP, Thompson SG, Deeks JJ, Altman DG. Measuring inconsistency in meta-analyses. BMJ. 2003;327(7414):557. doi: 10.1136/bmj.327.7414.557 12958120 PMC192859

[25] Sterne JA, Harbord RM. Funnel plots in meta-analysis. Stata J. 2004;4:127–41.

[26] Harbord RM, Higgins JP. Meta-regression in Stata. Stata J. 2008;8(4):493–519.

[27] Amrhein V, Greenland S, McShane B. Scientists rise up against statistical significance. Nature. 2019;567:305–7. doi: 10.1038/d41586-019-00857-9 30894741

[28] Gakidou E, Afshin A, Abajobir AA, Abate KH, Abbafati C, Abbas KM, et al. Global, regional, and national comparative risk assessment of 84 behavioural, environmental and occupational, and metabolic risks or clusters of risks, 1990–2016: a systematic analysis for the Global Burden of Disease Study 2016. Lancet. 2017;390(10100):1345–422. doi: 10.1016/S0140-6736(17)32366-8 28919119 PMC5614451

[29] Wylie BJ, Matechi E, Kishashu Y, Fawzi W, Premji Z, Coull BA, et al. Placental pathology associated with household air pollution in a cohort of pregnant women from Dar es Salaam, Tanzania. Environ Health Perspect. 2017;125(1):134–40. doi: 10.1289/EHP256 27286442 PMC5226703

[30] Shupler M, Godwin W, Frostad J, Gustafson P, Arku RE, Brauer M. Global estimation of exposure to fine particulate matter (PM2. 5) from household air pollution. Environ Int. 2018;120:354–63. doi: 10.1016/j.envint.2018.08.026 30119008

[31] Shaddick G, Thomas ML, Green A, Brauer M, Donkelaar A, Burnett R, et al. Data integration model for air quality: a hierarchical approach to the global estimation of exposures to ambient air pollution. J R Stat Soc Ser C Appl Stat. 2018;67(1):231–53.

[32] Trasande L, Malecha P, Attina TM. Particulate matter exposure and preterm birth: Estimates of US attributable burden and economic costs. Environ Health Perspect. 2016;124(12):1913. doi: 10.1289/ehp.1510810 27022947 PMC5132647

[33] Liu A, Qian N, Yu H, Chen R, Kan H. Estimation of disease burdens on preterm births and low birth weights attributable to maternal fine particulate matter exposure in Shanghai, China. Sci Total Environ 2017;609:815–21. Epub 2017 Aug 3. doi: 10.1016/j.scitotenv.2017.07.174 . 28768214

[34] Fleischer NL, Merialdi M, van Donkelaar A, Vadillo-Ortega F, Martin RV, Betran AP, et al. Outdoor Air Pollution, Preterm Birth, and Low Birth Weight: Analysis of the World Health Organization Global Survey on Maternal and Perinatal Health. Environ Health Perspect. 2014;122(4):425–30. doi: 10.1289/ehp.1306837 PMC3984219. 24508912 PMC3984219

[35] Bekkar B, Pacheco S, Basu R, DeNicola N. Association of Air Pollution and Heat Exposure With Preterm Birth, Low Birth Weight, and Stillbirth in the US: A Systematic Review. JAMA Netw Open. 2020;3(6):e208243. doi: 10.1001/jamanetworkopen.2020.8243 32556259 PMC7303808

[36] Liu C, Sun J, Liu Y, Liang H, Wang M, Wang C, et al. Different exposure levels of fine particulate matter and preterm birth: a meta-analysis based on cohort studies. Environ Sci Pollut Res. 2017;24(22):17976–84. doi: 10.1007/s11356-017-9363-0 28616740

[37] Parker JD, Mendola P, Woodruff TJ. Preterm birth after the Utah Valley Steel Mill closure: a natural experiment. Epidimiology. 2008;19(6):820–3. doi: 10.1097/EDE.0b013e3181883d5d 18854706

[38] Rich DQ, Liu K, Zhang J, Thurston SW, Stevens TP, Pan Y, et al. Differences in birth weight associated with the 2008 Beijing Olympics air pollution reduction: results from a natural experiment. Environ Health Perspect. 2015;123(9):880–7. doi: 10.1289/ehp.1408795 25919693 PMC4559955

[39] Wilcox AJ, Weinberg CR, Basso O. On the pitfalls of adjusting for gestational age at birth. Am J Epidemiol. 2011;174(9):1062–8. doi: 10.1093/aje/kwr230 21946386 PMC3243938

[40] Veras MM, Damaceno-Rodrigues NR, Caldini EG, Ribeiro AAM, Mayhew TM, Saldiva PH, et al. Particulate urban air pollution affects the functional morphology of mouse placenta. Biol Reprod. 2008;79(3):578–84. doi: 10.1095/biolreprod.108.069591 18509159

[41] Veras MM, Guimarães-Silva RM, Caldini EG, Saldiva PH, Dolhnikoff M, Mayhew TM. The effects of particulate ambient air pollution on the murine umbilical cord and its vessels: a quantitative morphological and immunohistochemical study. Reprod Toxicol. 2012;34(4):598–606. doi: 10.1016/j.reprotox.2012.08.003 22975478

[42] van den Hooven EH, Pierik FH, de Kluizenaar Y, Hofman A, van Ratingen SW, Zandveld PY, et al. Air pollution exposure and markers of placental growth and function: the generation R study. Environ Health Perspect. 2012;120(12):1753–9. doi: 10.1289/ehp.1204918 22922820 PMC3548279

[43] Janssen BG, Munters E, Pieters N, Smeets K, Cox B, Cuypers A, et al. Placental mitochondrial DNA content and particulate air pollution during in utero life. Environ Health Perspect. 2012;120(9):1346–52. doi: 10.1289/ehp.1104458 22626541 PMC3440109

[44] Nawrot TS, Saenen ND, Schenk J, Janssen BG, Motta V, Tarantini L, et al. Placental circadian pathway methylation and in utero exposure to fine particle air pollution. Environ Int. 2018;114:231–41. doi: 10.1016/j.envint.2018.02.034 29524919

[45] de Fátima SS, de Melo JO, Marchesi GDA, Lopes KL, Veras MM, de Oliveira IB, et al. Exposure to fine particulate matter in the air alters placental structure and the renin-angiotensin system. PLoS ONE. 2017;12(8):e0183314. doi: 10.1371/journal.pone.0183314 28820906 PMC5562329

[46] Hettfleisch K, Bernardes LS, Carvalho MA, Pastro LDM, Vieira SE, Saldiva SR, et al. Short-term exposure to urban air pollution and influences on placental vascularization indexes. Environ Health Perspect. 2017;125(4):753–9. doi: 10.1289/EHP300 27384326 PMC5381983

[47] Carpenter DO, Arcaro K, Spink DC. Understanding the human health effects of chemical mixtures. Environ Health Perspect. 2002;110(Suppl 1):25–42. doi: 10.1289/ehp.02110s125 11834461 PMC1241145

[48] Šrám RJ, Binková B, Dejmek J, Bobak M. Ambient air pollution and pregnancy outcomes: a review of the literature. Environ Health Perspect. 2005;113(4):375. doi: 10.1289/ehp.6362 15811825 PMC1278474

[49] Dejmek J, Solanský I, Benes I, Lenícek J, Srám RJ. The impact of polycyclic aromatic hydrocarbons and fine particles on pregnancy outcome. Environ Health Perspect. 2000;108(12):1159–64. doi: 10.1289/ehp.001081159 11133396 PMC1240197

[50] Blank F, Wehrli M, Lehmann A, Baum O, Gehr P, von Garnier C, et al. Macrophages and dendritic cells express tight junction proteins and exchange particles in an in vitro model of the human airway wall. Immunobiology. 2011;216(1–2):86–95. doi: 10.1016/j.imbio.2010.02.006 20362352

[51] Van den Hooven EH, de Kluizenaar Y, Pierik FH, Hofman A, Van Ratingen SW, Zandveld PY, et al. Chronic air pollution exposure during pregnancy and maternal and fetal C-reactive protein levels: the Generation R Study. Environ Health Perspect. 2012;120(5):746–51. doi: 10.1289/ehp.1104345 22306530 PMC3346784

[52] Latzin P, Frey U, Armann J, Kieninger E, Fuchs O, Röösli M, et al. Exposure to moderate air pollution during late pregnancy and cord blood cytokine secretion in healthy neonates. PLoS ONE. 2011;6(8):e23130. doi: 10.1371/journal.pone.0023130 21826232 PMC3149643

[53] Dubowsky SD, Suh H, Schwartz J, Coull BA, Gold DR. Diabetes, obesity, and hypertension may enhance associations between air pollution and markers of systemic inflammation. Environ Health Perspect. 2006;114(7):992–8. doi: 10.1289/ehp.8469 16835049 PMC1513328

[54] Kannan S, Misra DP, Dvonch JT, Krishnakumar A. Exposures to airborne particulate matter and adverse perinatal outcomes: a biologically plausible mechanistic framework for exploring potential effect modification by nutrition. Environ Health Perspect. 2006;114(11):1636–42. doi: 10.1289/ehp.9081 17107846 PMC1665414

[55] Proietti E, Röösli M, Frey U, Latzin P. Air pollution during pregnancy and neonatal outcome: a review. J Aerosol Med Pulm Drug Deliv. 2013;26(1):9–23. doi: 10.1089/jamp.2011.0932 22856675

[56] Wilhelm M, Ritz B. Local variations in CO and particulate air pollution and adverse birth outcomes in Los Angeles County, California, USA. Environ Health Perspect. 2005;113(9):1212–21. doi: 10.1289/ehp.7751 16140630 PMC1280404

[57] Pedersen M, Stayner L, Slama R, Sørensen M, Figueras F, Nieuwenhuijsen MJ, et al. Ambient air pollution and pregnancy-induced hypertensive disorders: a systematic review and meta-analysis. Hypertension. 2014;64:494–500. doi: 10.1161/HYPERTENSIONAHA.114.03545 24935943

[58] Misra DP. The effect of the pregnancy-induced hypertension on fetal growth: a review of the literature. Paediatr Perinat Epidemiol. 1996;10(3):244–63. doi: 10.1111/j.1365-3016.1996.tb00048.x 8822768

[59] Malley CS, Kuylenstierna JC, Vallack HW, Henze DK, Blencowe H. Ashmore MR. Preterm birth associated with maternal fine particulate matter exposure: a global, regional and national assessment. Environment international 2017; 101:173–182. https://www.sciencedirect.com/science/article/pii/S0160412016305992 28196630 10.1016/j.envint.2017.01.023

