# Correction: Ambient and household PM_2.5_ pollution and adverse perinatal outcomes: A meta-regression and analysis of attributable global burden for 204 countries and territories

**DOI:** 10.1371/journal.pmed.1003852

**Published:** 2021-11-02

**Authors:** Rakesh Ghosh, Kate Causey, Katrin Burkart, Sarah Wozniak, Aaron Cohen, Michael Brauer

The fourth author’s name is spelled incorrectly. The correct name is: Sarah Wozniak.
