# Supplementary material for: Ambient and household PM2.5 pollution and adverse perinatal outcomes: A meta-regression and analysis of attributable global burden for 204 countries and territories
Source: PLoS Med. 2021 Sep 28;18(9):e1003718. doi: 10.1371/journal.pmed.1003718 (PMC8478226; doi:10.1371/journal.pmed.1003718)
Supplement: S1 PRISMA Checklist — (DOCX) [file pmed.1003718.s001.docx]

| **Section and Topic** | **Item #** | **Checklist item** | **Location where item is reported** |
| --- | --- | --- | --- |
| **TITLE** | | |  |
| Title | 1 | Identify the report as a systematic review. | ***Title*** |
| **ABSTRACT** | | |  |
| Abstract | 2 | See the PRISMA 2020 for Abstracts checklist. | Abstract includes the information suggested in the checklist. |
| **INTRODUCTION** | | |  |
| Rationale | 3 | Describe the rationale for the review in the context of existing knowledge. | Introduction paragraphs 1 and 2 |
| Objectives | 4 | Provide an explicit statement of the objective(s) or question(s) the review addresses. | Last paragraph of introduction |
| **METHODS** | | |  |
| Eligibility criteria | 5 | Specify the inclusion and exclusion criteria for the review and how studies were grouped for the syntheses. | Methods paragraphs 3 and 4. Supplementary information, and Supplementary table 3 |
| Information sources | 6 | Specify all databases, registers, websites, organisations, reference lists and other sources searched or consulted to identify studies. Specify the date when each source was last searched or consulted. | Methods paragraphs 1 and 2. |
| Search strategy | 7 | Present the full search strategies for all databases, registers and websites, including any filters and limits used. | Supplementary information. |
| Selection process | 8 | Specify the methods used to decide whether a study met the inclusion criteria of the review, including how many reviewers screened each record and each report retrieved, whether they worked independently, and if applicable, details of automation tools used in the process. | Methods paragraphs 3 and 4. Supplementary information |
| Data collection process | 9 | Specify the methods used to collect data from reports, including how many reviewers collected data from each report, whether they worked independently, any processes for obtaining or confirming data from study investigators, and if applicable, details of automation tools used in the process. | Methods paragraphs 3 and 4. Supplementary information |
| Data items | 10a | List and define all outcomes for which data were sought. Specify whether all results that were compatible with each outcome domain in each study were sought (e.g. for all measures, time points, analyses), and if not, the methods used to decide which results to collect. | All outcomes are defined when it first occurred in the manuscript. |
|  | 10b | List and define all other variables for which data were sought (e.g. participant and intervention characteristics, funding sources). Describe any assumptions made about any missing or unclear information. | Supplementary table 3. |
| Study risk of bias assessment | 11 | Specify the methods used to assess risk of bias in the included studies, including details of the tool(s) used, how many reviewers assessed each study and whether they worked independently, and if applicable, details of automation tools used in the process. | Methods paragraphs 6 and 7. |
| Effect measures | 12 | Specify for each outcome the effect measure(s) (e.g. risk ratio, mean difference) used in the synthesis or presentation of results. | Methods paragraph 8. |
| Synthesis methods | 13a | Describe the processes used to decide which studies were eligible for each synthesis (e.g. tabulating the study intervention characteristics and comparing against the planned groups for each synthesis (item #5)). | Methods paragraphs 3 and 4. Supplementary information, and Supplementary table 3 |
|  | 13b | Describe any methods required to prepare the data for presentation or synthesis, such as handling of missing summary statistics, or data conversions. | Methods paragraphs 3 and 4. Supplementary information, |
|  | 13c | Describe any methods used to tabulate or visually display results of individual studies and syntheses. | Methods paragraph 8. Forest and funnel plots in supplementary information. |
|  | 13d | Describe any methods used to synthesize results and provide a rationale for the choice(s). If meta-analysis was performed, describe the model(s), method(s) to identify the presence and extent of statistical heterogeneity, and software package(s) used. | Methods paragraph 8 and the very last sentence of the methods section. |
|  | 13e | Describe any methods used to explore possible causes of heterogeneity among study results (e.g. subgroup analysis, meta-regression). | Methods paragraph 8 |
|  | 13f | Describe any sensitivity analyses conducted to assess robustness of the synthesized results. | Methods paragraph 5 |
| Reporting bias assessment | 14 | Describe any methods used to assess risk of bias due to missing results in a synthesis (arising from reporting biases). | Not applicable. Those studies were excluded. |
| Certainty assessment | 15 | Describe any methods used to assess certainty (or confidence) in the body of evidence for an outcome. | Methods paragraph 8 |
| **RESULTS** | | |  |
| Study selection | 16a | Describe the results of the search and selection process, from the number of records identified in the search to the number of studies included in the review, ideally using a flow diagram. | Results paragraph 1 and supplementary information flow chart |
|  | 16b | Cite studies that might appear to meet the inclusion criteria, but which were excluded, and explain why they were excluded. | Supplementary information section, reasons for exclusion. |
| Study characteristics | 17 | Cite each included study and present its characteristics. | Supplementary table 3 |
| Risk of bias in studies | 18 | Present assessments of risk of bias for each included study. | Supplementary table 3 |
| Results of individual studies | 19 | For all outcomes, present, for each study: (a) summary statistics for each group (where appropriate) and (b) an effect estimate and its precision (e.g. confidence/credible interval), ideally using structured tables or plots. | Results paragraph 2 |
| Results of syntheses | 20a | For each synthesis, briefly summarise the characteristics and risk of bias among contributing studies. | Results paragraph 2 |
|  | 20b | Present results of all statistical syntheses conducted. If meta-analysis was done, present for each the summary estimate and its precision (e.g. confidence/credible interval) and measures of statistical heterogeneity. If comparing groups, describe the direction of the effect. | Results subsection on mete-regressions and forest plots. |
|  | 20c | Present results of all investigations of possible causes of heterogeneity among study results. | Results subsection on mete-regressions and forest plots. |
|  | 20d | Present results of all sensitivity analyses conducted to assess the robustness of the synthesized results. | Results subsection on mete-regressions and forest plots. |
| Reporting biases | 21 | Present assessments of risk of bias due to missing results (arising from reporting biases) for each synthesis assessed. | Results subsection on mete-regressions and forest and funnel plots. |
| Certainty of evidence | 22 | Present assessments of certainty (or confidence) in the body of evidence for each outcome assessed. | Results subsection on mete-regressions and forest plots. |
| **DISCUSSION** | | |  |
| Discussion | 23a | Provide a general interpretation of the results in the context of other evidence. | Discussion paragraphs 1 and 2. |
|  | 23b | Discuss any limitations of the evidence included in the review. | Discussion paragraphs 3, 4, 5, 6 |
|  | 23c | Discuss any limitations of the review processes used. | Discussion paragraphs 3, 4, 5, 6 |
|  | 23d | Discuss implications of the results for practice, policy, and future research. | Overall discussion and conclusion |
| **OTHER INFORMATION** | | |  |
| Registration and protocol | 24a | Provide registration information for the review, including register name and registration number, or state that the review was not registered. | Not registered. Additionally, this study includes a global burden of disease assessment |
|  | 24b | Indicate where the review protocol can be accessed, or state that a protocol was not prepared. | Protocol was not prepared but a data extraction sheet was available, which is a supplement. |
|  | 24c | Describe and explain any amendments to information provided at registration or in the protocol. | Not applicable |
| Support | 25 | Describe sources of financial or non-financial support for the review, and the role of the funders or sponsors in the review. | Please see funding section. Funders had no role in the design or conduct of the study. |
| Competing interests | 26 | Declare any competing interests of review authors. | There is no conflict of interest. |
| Availability of data, code and other materials | 27 | Report which of the following are publicly available and where they can be found: template data collection forms; data extracted from included studies; data used for all analyses; analytic code; any other materials used in the review. | Data for the review is presented in supplementary table 3. IHME website has the exposure information for the global burden of disease analysis. |
