# Supplementary material for: Ambient and household PM2.5 pollution and adverse perinatal outcomes: A meta-regression and analysis of attributable global burden for 204 countries and territories
Source: PLoS Med. 2021 Sep 28;18(9):e1003718. doi: 10.1371/journal.pmed.1003718 (PMC8478226; doi:10.1371/journal.pmed.1003718)
Supplement: S1 Text — (DOCX) [file pmed.1003718.s002.docx]

**Ambient and household PM_2.5_ pollution and adverse perinatal outcomes: A meta-regression and analysis of attributable global burden for 204 countries and territories**

**Online Supplementary Information**

**Outdoor air pollution search strategy**

**Databases searched** – PubMed, Embase, Web of Science, Scopus, Current Contents, Global Health, Cochrane, Toxline, Canadian Research Index and reference lists of all relevant articles. We restricted the search to peer-reviewed published literature in English language as searching grey literature and other languages was beyond the scope of this review.

**Time period** – Any start dates up to April 4, 2021.

**Restrictions** – “Humans”, NOT smoking NOT cigarette NOT secondhand smoke NOT environmental tobacco smoke NOT review

**Table A. Medical Subject Heading (MeSH) search terms used for electronic searches of databases.**

| **Preterm birth terms** | **Birth weight terms** | **Ambient air pollution terms** | **Household air pollution terms** |
| --- | --- | --- | --- |
| - Premature birth - Preterm birth - PTB - Preterm delivery - PTD - Prematurity - Gestational age | - Birth weight - Birthweight - BW | - Air pollution - Particulate matter - Particulates - PM - PM10 - PM 10 - PM2.5 - PM 2.5 - Total suspended particulates - TSP - Respirable particulates | - Indoor air pollution - Bio mass - Biomass - Bio-mass - Wood - Charcoal - Coal - Solid fuel - Dung - smok* - pollut* - Fuel - cook* - Indoor air - Household air - iap - hap - particulate |
|  | - Low birth weight - LBW - Term low birth weight |  |  |

In table S1, terms in each row were searched individually and all terms within a column were combined using an “OR” operator. The searches between two columns were then combined with “AND” operator.

**Data extraction** – A preformatted spreadsheet was developed with the members from IHME at the University of Washington, Seattle for data extraction from the individual studies.

**Fig A.** Flow diagram showing study selection in the systematic review and meta-regression for ambient air pollution

1400 potentially relevant citations were initially identified from MeSH search

984 were found unrelated to the aim after screening the titles

Abstracts were reviewed for 416 articles

215 were unrelated to the aim of the review

Full texts were reviewed for 201 articles

21 did not report any one of the three outcomes of interest

180 articles were included in the systematic review

Detailed reasons for exclusions are stated below

44 articles on birth weight and 40 on low birth weight and 40 on preterm birth were included in the meta-regression

**Fig B.** Flow diagram showing study selection in the systematic review and meta-regression for the household air pollution

190 citations were retrieved from MeSH search

157 were found unrelated to the aim after screening the titles and abstracts

Full texts were reviewed for 33 articles

13 did not report either the exposure or the outcome

11 were not case-control, cohort, or intervention studies

9 articles included in the systematic review

8 articles on birth weight, 6 on low birth weight, 3 on preterm birth, and 1 on gestational age were included in the meta-regression

**Fig C.** Meta-regression Bayesian Regularized and Trimmed models (solid black line) and 95% UIs (shaded region) for birth weight (a), gestational age (b), low birth weight (c) and preterm birth (d). The dots represent the point estimates, and the horizontal lines are the 5^th^ and the 95^th^ percentiles of the exposures from the original studies (horizontal lines, green: outdoor, red: household). The section of the curve outlined by the blue rectangle is expanded in the inset with the red rectangle representing the theoretical minimum risk exposure level.


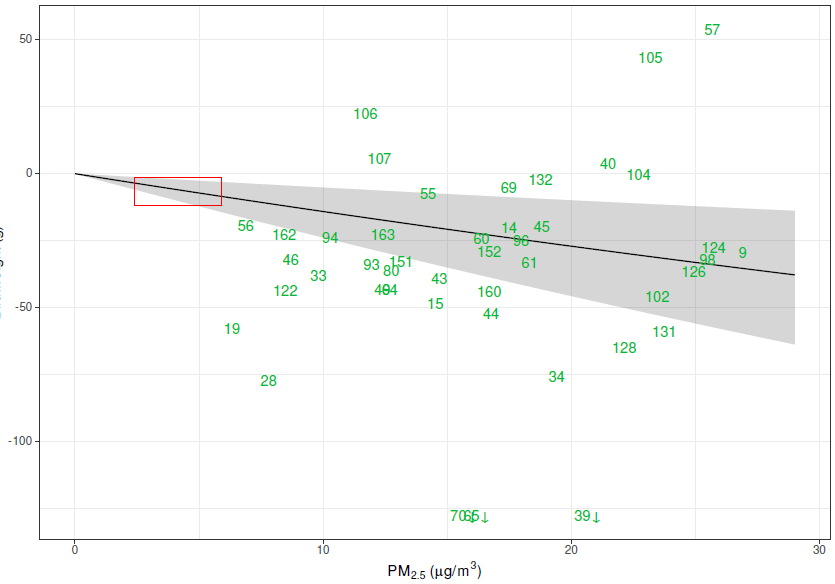

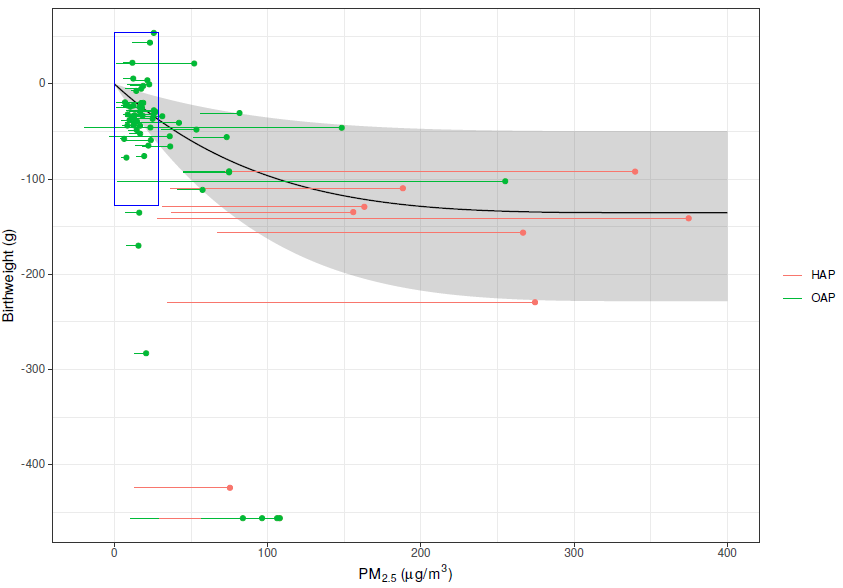


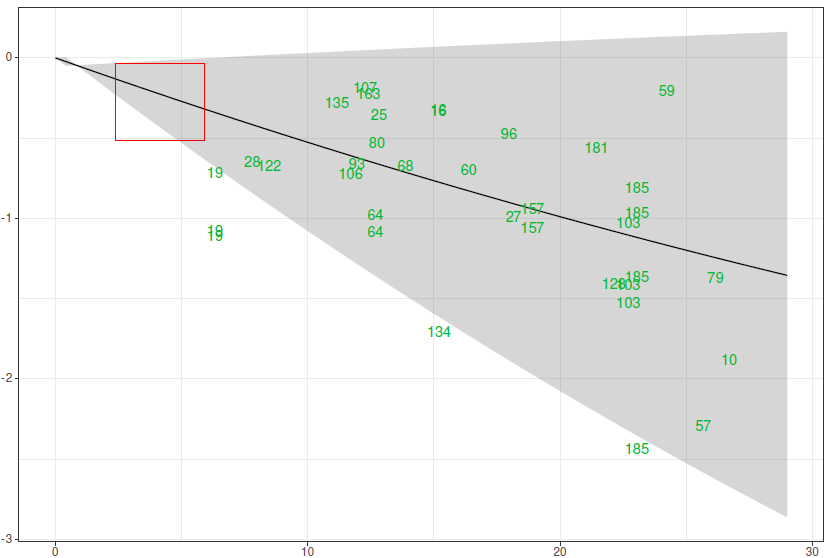

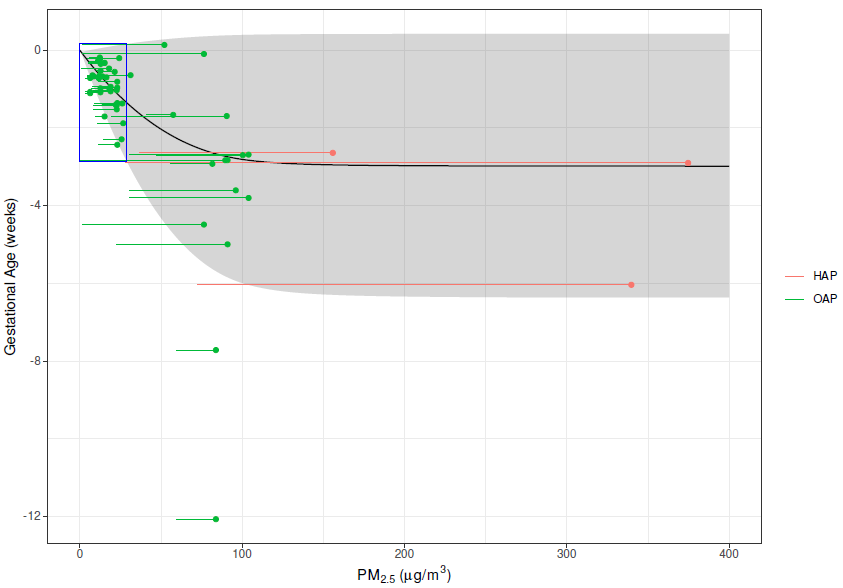


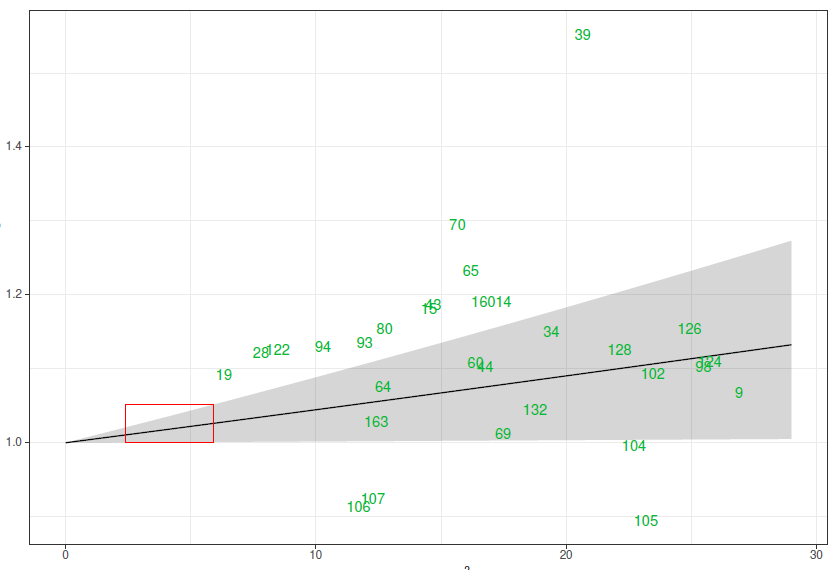

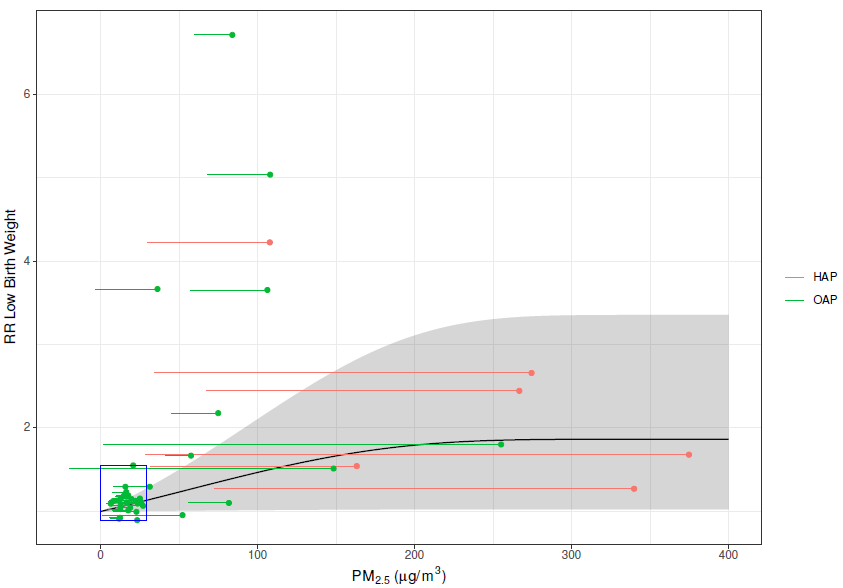


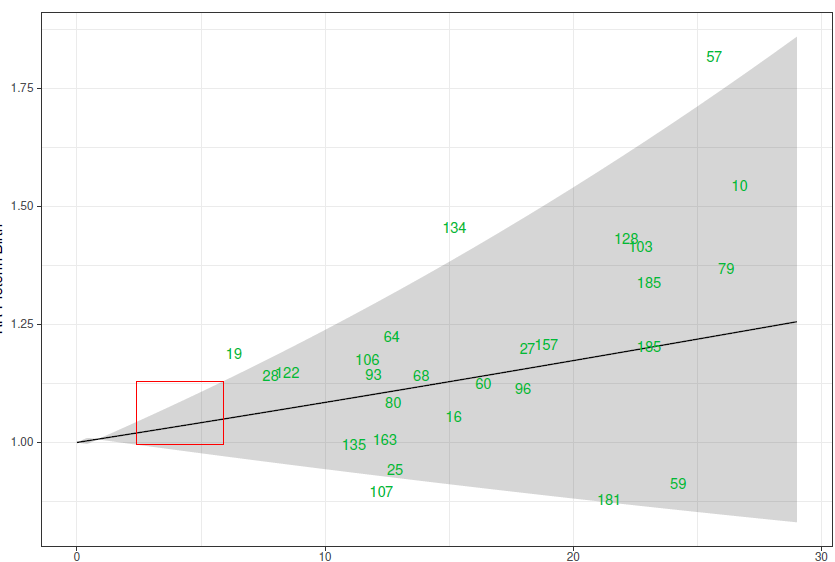

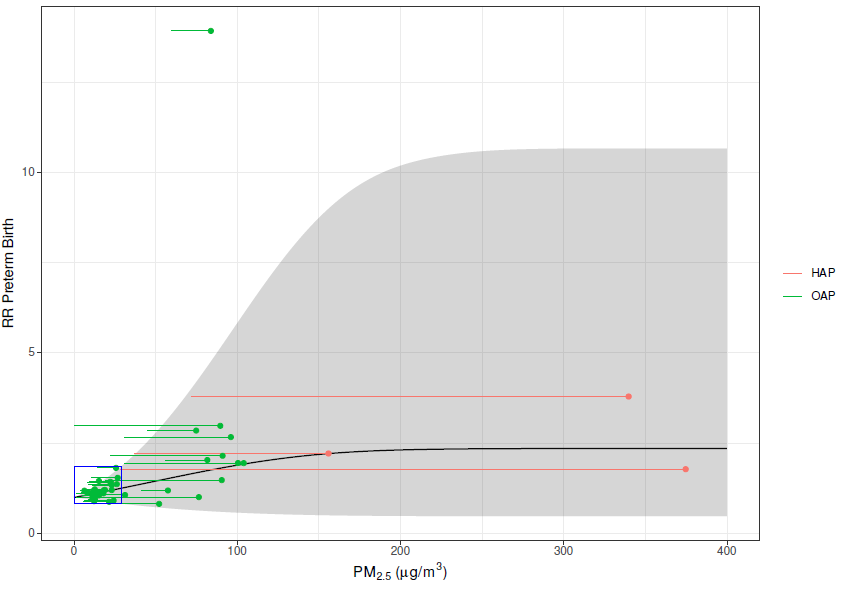


**Fig D.** Forest plots for birth weight (a), low birth weight (b), preterm birth (c) and ambient PM_2.5_

**(a)**

**(b)**

**(c)**

**Fig E.** Funnel plots for birth weight (a), low birth weight (b), preterm birth (c) and outdoor PM_2.5_.

**(a)**

**(b)**

**(c)**

**Fig F.** Forest plots for birth weight (a), low birth weight (b) and preterm birth (c) and household air pollution

**
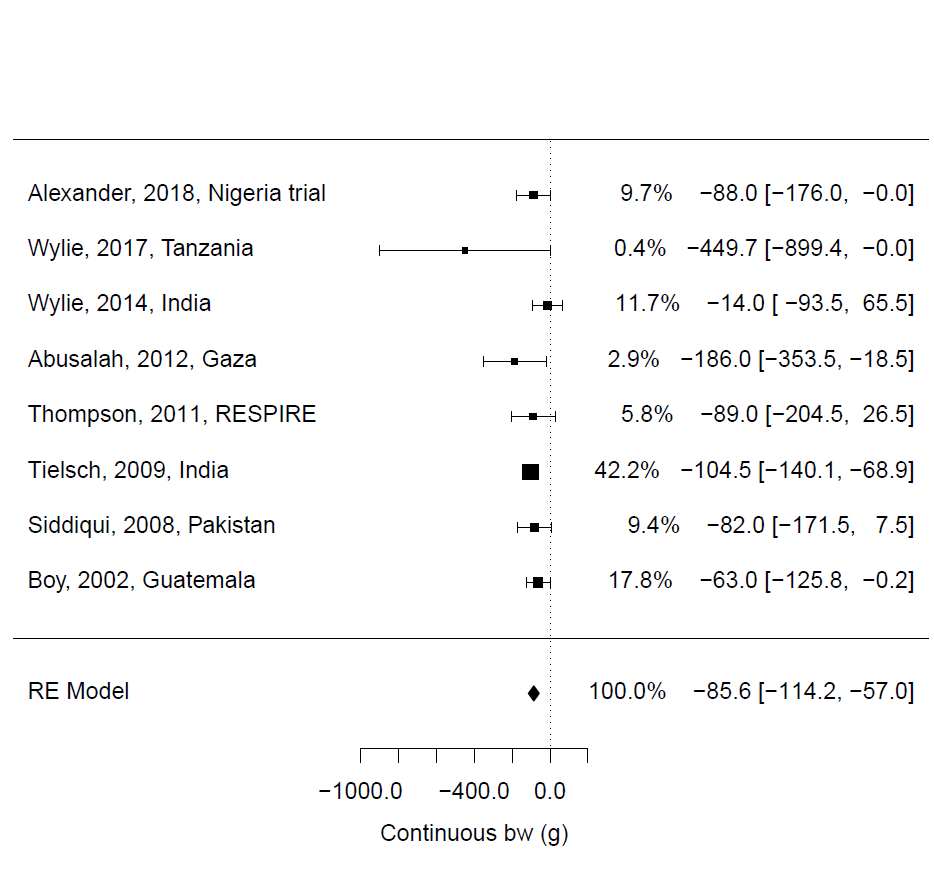
**

**(a)**


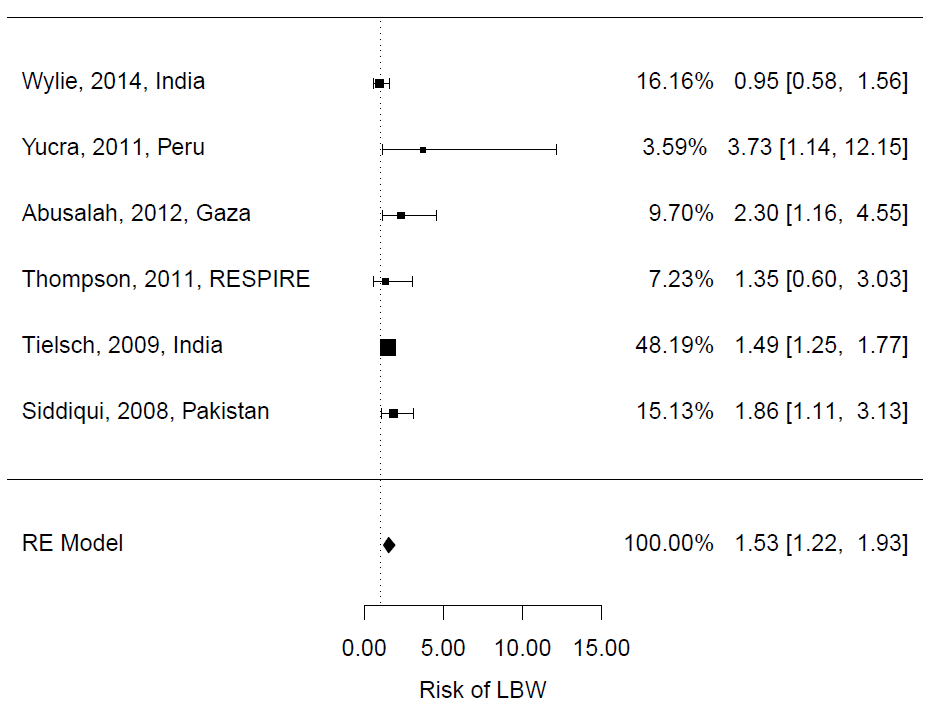


**(b)**

**
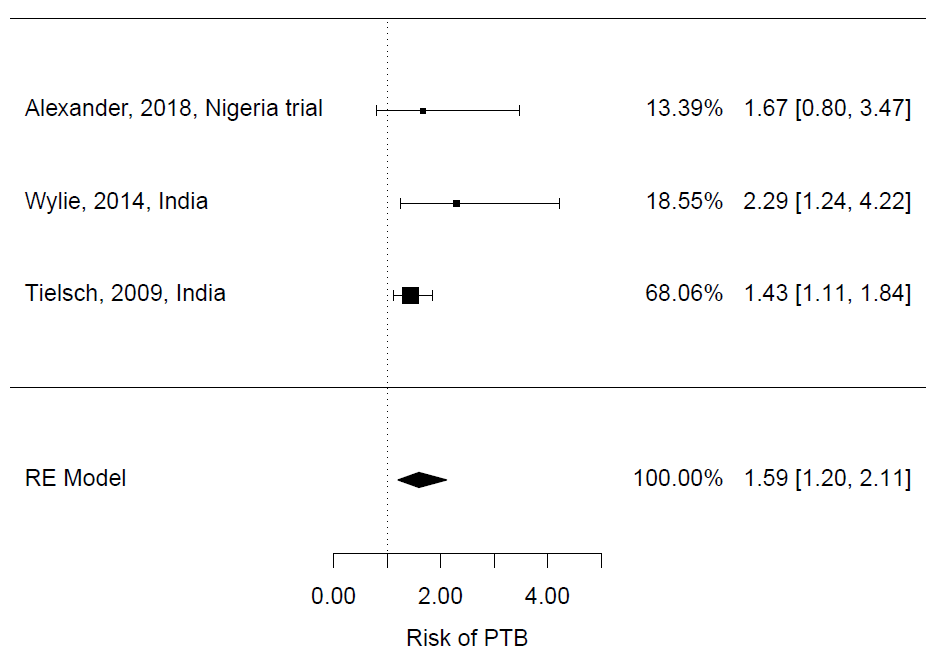
**

**(c)**
